# The *BRCA2* c.68‐7T > A variant is not pathogenic: A model for clinical calibration of spliceogenicity

**DOI:** 10.1002/humu.23411

**Published:** 2018-04-06

**Authors:** Mara Colombo, Irene Lòpez‐Perolio, Huong D. Meeks, Laura Caleca, Michael T. Parsons, Hongyan Li, Giovanna De Vecchi, Emma Tudini, Claudia Foglia, Patrizia Mondini, Siranoush Manoukian, Raquel Behar, Encarna B. Gómez Garcia, Alfons Meindl, Marco Montagna, Dieter Niederacher, Ane Y. Schmidt, Liliana Varesco, Barbara Wappenschmidt, Manjeet K. Bolla, Joe Dennis, Kyriaki Michailidou, Qin Wang, Kristiina Aittomäki, Irene L. Andrulis, Hoda Anton‐Culver, Volker Arndt, Matthias W. Beckmann, Alicia Beeghly‐Fadel, Javier Benitez, Bram Boeckx, Natalia V. Bogdanova, Stig E. Bojesen, Bernardo Bonanni, Hiltrud Brauch, Hermann Brenner, Barbara Burwinkel, Jenny Chang‐Claude, Don M. Conroy, Fergus J. Couch, Angela Cox, Simon S. Cross, Kamila Czene, Peter Devilee, Thilo Dörk, Mikael Eriksson, Peter A. Fasching, Jonine Figueroa, Olivia Fletcher, Henrik Flyger, Marike Gabrielson, Montserrat García‐Closas, Graham G. Giles, Anna González‐Neira, Pascal Guénel, Christopher A. Haiman, Per Hall, Ute Hamann, Mikael Hartman, Jan Hauke, Antoinette Hollestelle, John L. Hopper, Anna Jakubowska, Audrey Jung, Veli‐Matti Kosma, Diether Lambrechts, Loid Le Marchand, Annika Lindblom, Jan Lubinski, Arto Mannermaa, Sara Margolin, Hui Miao, Roger L. Milne, Susan L. Neuhausen, Heli Nevanlinna, Janet E. Olson, Paolo Peterlongo, Julian Peto, Katri Pylkäs, Elinor J. Sawyer, Marjanka K. Schmidt, Rita K. Schmutzler, Andreas Schneeweiss, Minouk J. Schoemaker, Mee Hoong See, Melissa C. Southey, Anthony Swerdlow, Soo H. Teo, Amanda E. Toland, Ian Tomlinson, Thérèse Truong, Christi J. van Asperen, Ans M.W. van den Ouweland, Lizet E. van der Kolk, Robert Winqvist, Drakoulis Yannoukakos, Wei Zheng, kConFab/AOCS Investigators, Alison M. Dunning, Douglas F. Easton, Alex Henderson, Frans B.L. Hogervorst, Louise Izatt, Kenneth Offitt, Lucy E. Side, Elizabeth J. van Rensburg, Study EMBRACE, Study HEBON, Lesley McGuffog, Antonis C. Antoniou, Georgia Chenevix‐Trench, Amanda B. Spurdle, David E. Goldgar, Miguel de la Hoya, Paolo Radice

**Affiliations:** ^1^ Unit of Molecular Bases of Genetic Risk and Genetic Testing Department of Research Fondazione IRCCS (Istituto di Ricovero e Cura a Carattere Scientifico) Istituto Nazionale dei Tumori (INT) Milan Italy; ^2^ Molecular Oncology Laboratory CIBERONC Hospital Clinico San Carlos IdISSC (Instituto de Investigación Sanitaria del Hospital Clínico San Carlos) Madrid Spain; ^3^ Huntsman Cancer Institute University of Utah Salt Lake City Utah; ^4^ Department of Genetics and Computational Biology QIMR Berghofer Medical Research Institute Brisbane, QLD 4006 Australia; ^5^ Unit of Medical Genetics Department of Medical Oncology and Hematology Fondazione IRCCS (Istituto di Ricovero e Cura a Carattere Scientifico) Istituto Nazionale dei Tumori (INT) Milan Italy; ^6^ Department of Clinical Genetics and GROW School for Oncology and Developmental Biology MUMC Maastricht The Netherlands; ^7^ Department of Obstetrics and Gynecology University Hospital LMU Munich Germany; ^8^ Immunology and Molecular Oncology Unit Veneto Institute of Oncology IOV ‐ IRCCS Padua Italy; ^9^ Department of Gynaecology and Obstetrics University Hospital Düsseldorf Heinrich‐Heine University Duesseldorf Germany; ^10^ Center for Genomic Medicine Rigshospitalet University of Copenhagen Copenhagen Denmark; ^11^ Hereditary Cancer Unit IRCCS AOU San Martino ‐IST Genova Italy; ^12^ Center for Hereditary Breast and Ovarian Cancer University Hospital of Cologne Cologne Germany; ^13^ Center for Integrated Oncology (CIO) Medical Faculty University Hospital of Cologne Cologne Germany; ^14^ Centre for Cancer Genetic Epidemiology Department of Public Health and Primary Care University of Cambridge Cambridge UK; ^15^ Department of Electron Microscopy/Molecular Pathology The Cyprus Institute of Neurology and Genetics Nicosia Cyprus; ^16^ Department of Clinical Genetics Helsinki University Hospital University of Helsinki Helsinki Finland; ^17^ Fred A. Litwin Center for Cancer Genetics Lunenfeld‐Tanenbaum Research Institute of Mount Sinai Hospital Toronto Ontario; ^18^ Department of Molecular Genetics University of Toronto Toronto Canada; ^19^ Department of Epidemiology University of California Irvine Irvine California; ^20^ Division of Clinical Epidemiology and Aging Research German Cancer Research Center (DKFZ) Heidelberg Germany; ^21^ Department of Gynaecology and Obstetrics University Hospital Erlangen, Friedrich‐Alexander University Erlangen‐Nuremberg Comprehensive Cancer Center Erlangen‐EMN Erlangen Germany; ^22^ Division of Epidemiology Department of Medicine Vanderbilt Epidemiology Center Vanderbilt‐Ingram Cancer Center Vanderbilt University School of Medicine Nashville Tennessee; ^23^ Human Cancer Genetics Program Spanish National Cancer Research Centre Madrid Spain; ^24^ Centro de Investigación en Red de Enfermedades Raras (CIBERER) Valencia Spain; ^25^ VIB Center for Cancer Biology VIB Leuven Belgium; ^26^ Laboratory for Translational Genetics Department of Human Genetics University of Leuven Leuven Belgium; ^27^ Department of Radiation Oncology Hannover Medical School Hannover Germany; ^28^ Gynaecology Research Unit Hannover Medical School Hannover Germany; ^29^ N.N. Alexandrov Research Institute of Oncology and Medical Radiology Minsk Belarus; ^30^ Copenhagen General Population Study Herlevand Gentofte Hospital Copenhagen University Hospital Herlev Denmark; ^31^ Department of Clinical Biochemistry Herlev and Gentofte Hospital Copenhagen University Hospital Herlev Denmark; ^32^ Faculty of Health and Medical Sciences University of Copenhagen Copenhagen Denmark; ^33^ Division of Cancer Prevention and Genetics Istituto Europeo di Oncologia Milan Italy; ^34^ Dr. Margarete Fischer‐Bosch‐Institute of Clinical Pharmacology Stuttgart Germany; ^35^ University of Tübingen Tübingen Germany; ^36^ German Cancer Consortium (DKTK) German Cancer Research Center (DKFZ) Heidelberg Germany; ^37^ Division of Preventive Oncology German Cancer Research Center (DKFZ) and National Center for Tumor Diseases (NCT) Heidelberg Germany; ^38^ Department of Obstetrics and Gynecology University of Heidelberg Heidelberg Germany; ^39^ Molecular Epidemiology Group C080 German Cancer Research Center (DKFZ) Heidelberg Germany; ^40^ Division of Cancer Epidemiology German Cancer Research Center (DKFZ) Heidelberg Germany; ^41^ Research Group Genetic Cancer Epidemiology University Cancer Center Hamburg (UCCH) University Medical Center Hamburg‐Eppendorf Hamburg Germany; ^42^ Centre for Cancer Genetic Epidemiology Department of Oncology University of Cambridge Cambridge UK; ^43^ Department of Laboratory Medicine and Pathology Mayo Clinic Rochester New York; ^44^ Sheffield Institute for Nucleic Acids (SInFoNiA) Department of Oncology and Metabolism University of Sheffield Sheffield UK; ^45^ Academic Unit of Pathology Department of Neuroscience University of Sheffield Sheffield UK; ^46^ Department of Medical Epidemiology and Biostatistics Karolinska Institutet Stockholm Sweden; ^47^ Department of Pathology Leiden University Medical Center Leiden The Netherlands; ^48^ Department of Human Genetics Leiden University Medical Center Leiden The Netherlands; ^49^ David Geffen School of Medicine Department of Medicine Division of Hematology and Oncology University of California at Los Angeles Los Angeles California; ^50^ Usher Institute of Population Health Sciences and Informatics The University of Edinburgh Medical School Edinburgh UK; ^51^ Division of Cancer Epidemiology and Genetics National Cancer Institute Rockville Maryland; ^52^ The Breast Cancer Now Toby Robins Research Centre The Institute of Cancer Research London UK; ^53^ Department of Breast Surgery Herlev and Gentofte Hospital Copenhagen University Hospital Herlev Denmark; ^54^ Cancer Epidemiology & Intelligence Division Cancer Council Victoria Melbourne Australia; ^55^ Centre for Epidemiology and Biostatistics Melbourne School of Population and Global health The University of Melbourne Melbourne Australia; ^56^ Cancer & Environment Group Center for Research in Epidemiology and Population Health (CESP) INSERM University Paris‐Sud University Paris‐Saclay Villejuif France; ^57^ Department of Preventive Medicine Keck School of Medicine University of Southern California Los Angeles California; ^58^ Molecular Genetics of Breast Cancer Deutsches Krebsforschungszentrum (DKFZ) Heidelberg Germany; ^59^ Saw Swee Hock School of Public Health National University of Singapore Singapore Singapore; ^60^ Department of Surgery National University Health System Singapore Singapore; ^61^ Center for Molecular Medicine Cologne (CMMC) University of Cologne Cologne Germany; ^62^ Department of Medical Oncology Family Cancer Clinic Erasmus MC Cancer Institute Rotterdam The Netherlands; ^63^ Department of Genetics and Pathology Pomeranian Medical University Szczecin Poland; ^64^ Translational Cancer Research Area University of Eastern Finland Kuopio Finland; ^65^ Institute of Clinical Medicine Pathology and Forensic Medicine University of Eastern Finland Kuopio Finland; ^66^ Imaging Center Department of Clinical Pathology Kuopio University Hospital Kuopio Finland; ^67^ Epidemiology Program University of Hawaii Cancer Center Honolulu Hawaii; ^68^ Department of Molecular Medicine and Surgery Karolinska Institutet Stockholm Sweden; ^69^ Department of Clinical Science and Education Södersjukhuset Karolinska Institutet Stockholm Sweden; ^70^ Department of Population Sciences Beckman Research Institute of City of Hope Duarte California; ^71^ Department of Obstetrics and Gynecology Helsinki University Hospital University of Helsinki Helsinki Finland; ^72^ Department of Health Sciences Research Mayo Clinic Rochester New York; ^73^ IFOM The FIRC (Italian Foundation for Cancer Research) Institute of Molecular Oncology Milan Italy; ^74^ Department of Non‐Communicable Disease Epidemiology London School of Hygiene and Tropical Medicine London UK; ^75^ Laboratory of Cancer Genetics and Tumor Biology Cancer and Translational Medicine Research Unit Biocenter Oulu University of Oulu Oulu Finland; ^76^ Laboratory of Cancer Genetics and Tumor Biology Northern Finland Laboratory Centre Oulu Oulu Finland; ^77^ Research Oncology Guy's Hospital King's College London London UK; ^78^ Division of Molecular Pathology The Netherlands Cancer Institute – Antoni van Leeuwenhoek Hospital Amsterdam The Netherlands; ^79^ Division of Psychosocial Research and Epidemiology The Netherlands Cancer Institute – Antoni van Leeuwenhoek hospital Amsterdam The Netherlands; ^80^ National Center for Tumor Diseases University of Heidelberg Heidelberg Germany; ^81^ Division of Genetics and Epidemiology The Institute of Cancer Research London UK; ^82^ Breast Cancer Research Unit Cancer Research Institute University Malaya Medical Centre Kuala Lumpur Malaysia; ^83^ Department of Pathology The University of Melbourne Melbourne Australia; ^84^ Division of Breast Cancer Research The Institute of Cancer Research London UK; ^85^ Cancer Research Malaysia Subang Jaya Selangor Malaysia; ^86^ Department of Molecular Virology Immunology and Medical Genetics Comprehensive Cancer Center The Ohio State University Columbus Ohio; ^87^ Wellcome Trust Centre for Human Genetics and Oxford NIHR Biomedical Research Centre University of Oxford Oxford UK; ^88^ Department of Clinical Genetics Leiden University Medical Center Leiden The Netherlands; ^89^ Department of Clinical Genetics Erasmus University Medical Center Rotterdam The Netherlands; ^90^ Family Cancer Clinic The Netherlands Cancer Institute ‐ Antoni van Leeuwenhoek hospital Amsterdam The Netherlands; ^91^ Molecular Diagnostics Laboratory INRASTES National Centre for Scientific Research “Demokritos” Athens Greece; ^92^ Peter MacCallum Cancer Center Melbourne Australia; ^93^ Institute of Genetic Medicine Centre for Life Newcastle Upon Tyne Hospitals NHS Trust Newcastle upon Tyne UK; ^94^ Clinical Genetics Guy's and St. Thomas’ NHS Foundation Trust London UK; ^95^ Clinical Genetics Research Laboratory Dept. of Medicine Cancer Biology and Genetics Memorial Sloan‐Kettering Cancer Center New York New York; ^96^ Wessex Clinical Genetics Service Mailpoint 627, Princess Anne Hospital, Coxford Road, Southampton, SO16 5YA; ^97^ Cancer Genetics Laboratory Department of Genetics University of Pretoria Pretoria South Africa; ^98^ Centre for Cancer Genetic Epidemiology Department of Public Health and Primary Care University of Cambridge Strangeways Research Laboratory Worts Causeway Cambridge UK; ^99^ The Hereditary Breast and Ovarian Cancer Research Group Netherlands (HEBON) Coordinating center: Netherlands Cancer Institute Amsterdam The Netherlands; ^100^ Centre for Cancer Genetic Epidemiology Department of Public Health and Primary Care University of Cambridge Cambridge UK

**Keywords:** *BRCA2*, digital PCR, multifactorial likelihood analysis, quantitative real‐time PCR, spliceogenic variants

## Abstract

Although the spliceogenic nature of the *BRCA2* c.68‐7T > A variant has been demonstrated, its association with cancer risk remains controversial. In this study, we accurately quantified by real‐time PCR and digital PCR (dPCR), the *BRCA2* isoforms retaining or missing exon 3. In addition, the combined odds ratio for causality of the variant was estimated using genetic and clinical data, and its associated cancer risk was estimated by case‐control analysis in 83,636 individuals. Co‐occurrence in trans with pathogenic *BRCA2* variants was assessed in 5,382 families. Exon 3 exclusion rate was 4.5‐fold higher in variant carriers (13%) than controls (3%), indicating an exclusion rate for the c.68‐7T > A allele of approximately 20%. The posterior probability of pathogenicity was 7.44 × 10^−115^. There was neither evidence for increased risk of breast cancer (OR 1.03; 95% CI 0.86–1.24) nor for a deleterious effect of the variant when co‐occurring with pathogenic variants. Our data provide for the first time robust evidence of the nonpathogenicity of the *BRCA2* c.68‐7T > A. Genetic and quantitative transcript analyses together inform the threshold for the ratio between functional and altered *BRCA2* isoforms compatible with normal cell function. These findings might be exploited to assess the relevance for cancer risk of other *BRCA2* spliceogenic variants.

## 1 INTRODUCTION

*BRCA1* (MIM# 113705) and *BRCA2* (MIM# 600185) are tumor suppressor genes and their inactivation promotes cancer development. Carriers of germline pathogenic variants in these genes are at high risk of developing breast and ovarian cancers, and *BRCA1/2* gene testing has become a widely used procedure in the clinical management of families suspected of hereditary susceptibility to these malignancies. The individuals within these families, identified as at‐risk based on their genetic profile, may benefit from risk‐reduction options. However, the usefulness of genetic testing relies on the ability to ascertain the pathogenic nature of the identified genetic variants, which is not necessarily straightforward for small in‐frame deletions and insertions, variants in regulatory sequences, missense, synonymous and intronic changes, and variants introducing premature protein‐truncating codons at the 3′ end of the coding sequence.

The Evidence‐based Network for the Interpretation of Germline Mutant Alleles (ENIGMA) has developed and documented criteria aimed at determining the clinical significance of sequence variants in BRCA genes (https://www.enigmaconsortium.org). The classification, based on a five‐class system (Plon et al., 2008), is intended to differentiate high risk variants (risk equivalent to that of protein‐truncating pathogenic variants), including pathogenic and likely pathogenic variants (class 5 and 4, respectively), from variants with low or no risk, including not pathogenic and likely not pathogenic variants (class 1 and 2, respectively). Variants for which clinical significance is unclear are placed in class 3 and are referred to as variants of uncertain significance (VUSs).

One controversial variant in *BRCA2* is c.68‐7T > A, which lies upstream of the acceptor splice site of exon 3. This variant (rs81002830) has been reported in several populations worldwide with an allelic frequency ranging from 0.02% in East Asians to 0.5% in non‐Finnish Europeans (Lek et al., 2016). Several authors have reported c.68‐7T > A being spliceogenic, that is, able to alter normal premRNA splicing. In particular, using semiquantitative approaches, it has been documented that the variant leads to an increase of the naturally occurring transcripts lacking exon 3 (∆3) (Houdayer et al., 2012; Jarhelle, Riise Stensland, Maehle, & Van Ghelue, 2016; Sanz et al., 2010; Thery et al., 2011; Vreeswijk et al., 2009). A competitive quantitative PCR (qPCR) analysis estimated that the proportion of the ∆3 transcript compared to full length was approximately 25% in variant samples versus 4% in normal samples (Muller et al., 2011). More recently, segregation analyses in two families indicated that the variant did not segregate in the affected branches (Santos et al., 2014). Although a few of the above studies tentatively classified the variant as benign or likely benign, they do not provide robust genetic evidence to justify this conclusion. Conversely, a recent article asserted that the variant was associated with breast cancer, based on a relatively limited case control association study in the Norwegian population (Møller & Hovig, 2017).

As a consequence, to date the classification of c.68‐7T > A reported in databases aggregating information on genomic variations has remained inconclusive. In particular, ClinVar (https://www.ncbi.nlm.nih.gov/clinvar/, last updated: Feb 1, 2018) reports conflicting interpretations classifying the variant as benign (seven entries), likely benign (nine entries) and of uncertain significance (four entries). Moreover, the BIC (Breast Cancer Information Core, https://research.nhgri.nih.gov/bic/) database presently annotates the variant as of unknown clinical importance, pending classification, while the BRCA Share™ (UMD‐BRCA2 mutations database) (https://www.umd.be/BRCA2/) classifies it as likely benign.

In the present study, we combined genetic approaches, including a large multicentre case‐control study and segregation analysis in a sizable number of families, with qualitative and quantitative analyses of the transcripts, and Mitomycin C growth inhibition test. Our findings provide a robust classification of the *BRCA2* c.68‐7T > A variant with respect to its effect on cancer risk, and add evidence that splicing pattern alterations do not necessarily lead to pathogenicity.

## 2 MATERIALS AND METHODS

### 2.1 Nomenclature

The nucleotide numbering was based on the reference *BRCA2* complementary deoxyribonucleic acid (cDNA) sequence NM_000059.3. For the purposes of the study, we defined as ▼3 all *BRCA2* isoforms retaining exon 3 and as ∆3 all *BRCA2* isoforms missing exon 3, irrespective of additional alternative splicing events.

### 2.2 Cell lines

Epstein‐Barr virus (EBV)‐immortalized human lymphoblastoid cell lines (LCLs) were obtained as previously described (Colombo et al., 2013). In this analysis 18 LCLs were considered, including six LCLs obtained from women carrying the *BRCA2* c.68‐7T > A variant and 12 LCLs obtained from healthy female blood donors, recruited at the Istituto Nazionale dei Tumori (INT) of Milan. The c.68‐7T > A carriers had been screened in all coding exons and corresponding intron‐exon junctions of both *BRCA1* and *BRCA2*. Excluding common polymorphisms, none of them carried additional BRCA gene variants, with a single exception where a protein‐truncating variant was detected in *BRCA1* (c.1380dupA). Only *BRCA2* exon 3 was sequenced in the LCLs from normal controls and no pathogenic variants or VUS were observed. The two *BRCA2*‐deficient cell lines, EUFA423 immortalized fibroblasts (*BRCA2^7691insAT/9900insA^*) (Howlett et al., 2002) and pancreatic cancer cell line Capan1 (*BRCA2^−/6174delT^*) (Goggins et al., 1996) were cultured as described elsewhere (Feng et al., 2011).

### 2.3 Cytoplasmic RNA isolation and first strand cDNA synthesis

Cytoplasmic RNA was isolated from fresh LCLs using the Cytoplasmic & Nuclear RNA Purification Kit (NORGEN BIOTEK CORPORATION, Canada), including the DNase I treatment according to manufacturer's instructions. The RNA purity and integrity was verified by measuring the A_260_/A_280_ ratio and by electrophoresis on agarose gel. For capillary electrophoresis (CE), allele‐specific expression analysis and reverse transcriptase quantitative PCR (RT‐qPCR), first strand cDNA was generated using 1 μg RNA, random hexamer primers and Maxima™ H Minus RT (Thermo Scientific), following the manufacturer's protocol in a final volume of 20 μl. For digital PCR (dPCR), 1 μg RNA was reverse transcribed with Prime‐Script RT kit (TaKaRa Biotechnology, Japan) according to the manufacturer's protocol using a mixture of random and Oligo (dT) primers. No‐RT controls, containing all reagents for the reverse transcription but the enzyme, were carried out.

### 2.4 Capillary electrophoresis analysis

Multiplex fluorescently‐labeled PCRs were performed with primers located upstream and downstream of exon 3, to simultaneously amplify both ▼3 and ∆3 transcripts, followed by CE analysis. A beta‐2‐microglobulin (*B2M;* MIM# 109700) cDNA fragment of 377 bp was co‐amplified to normalize CE peaks and allow comparison between cases and controls. The sequences of the primers are listed in Supp. Table S1. PCR amplifications were performed in 20 μl reaction volume containing 2 μl of cDNA solution under end‐point conditions. Cycling conditions were as follows: 95°C for 7 min, followed by 35 cycles at 95°C for 30′′, 58°C for 30′′ and 72°C for 30′′. A final 7 min elongation step was performed at 72°C. The fluorescent amplification products were run on an ABI 3130 Genetic Analyzer (Applied Biosystems). GeneScan™ 500 ROX™ dye size standard was used as internal size‐standard and size calling was performed with GeneMapper software v4.0 (Applied Biosystems).

### 2.5 Assessment of allelic expression of ▼3 and ∆3 transcripts

The allelic origin of the ▼3 and ∆3 transcripts were ascertained by amplification and sequencing of the region containing the common c.‐26G > A variant (rs1799943) in the 5′‐UTR of *BRCA2*. PCR reactions were performed as described above. The forward primer was designed to anneal to a region upstream of c.‐26G > A and the reverse primers to sequences in exon 3 and across the exon2‐exon4 junction, specific of the ▼3 and ∆3 transcripts, respectively (Supp. Table S1). Sequencing conditions were as previously described (Colombo et al., 2013).

### 2.6 Quantitative PCR analysis

Specific quantitative assays were designed to capture the expression levels of the ▼3 and Δ3 transcripts. The primer sets (Supp. Table S1) were validated with end‐point PCR reactions, and the specificity of the amplification products were confirmed by sequencing.

The qPCR analysis were performed on the Eco real‐time PCR system (Illumina) using SYBR^®^ Green I dye chemistry (KAPA SYBR^®^ FAST qPCR Kit, Kapa Biosystems). All reactions were carried out in a final volume of 10 μl containing 1 μl of cDNA and 200 nM of *GUSB* and ▼3 transcript specific primers, and 300 nM of Δ3 transcript specific primers. The efficiency of qPCR assays was evaluated based on a relative standard curve, using threefold serial dilutions starting from pooled control cDNAs in triplicate. The thermal profile was the same for all assays (95°C for 3 min, followed by 40 cycles of 95°C for 3 sec and 62°C for 20 sec). The melting curve analysis was performed according to default conditions (95°C for 15 sec, 55°C for 15 sec and 95°C for 15 sec). All samples from both cases and controls were individually analyzed in triplicate, and the corresponding average values were considered. No template controls and no‐RT controls were included in the analysis. The data, obtained in the form of quantification cycle (Cq), were normalized using the beta‐glucuronidase gene (*GUSB*) (de Brouwer, van Bokhoven, & Kremer, 2006). The obtained values were used to compute, in both normal and mutated samples, *BRCA2* exon 3 exclusion rate, that is, the percentage of *BRCA2* mRNA isoforms missing exon 3 over the total amount of *BRCA2* transcripts, as follows:

$$[2^{-\Delta Cq_{\Delta 3}}/\left(2^{-\Delta Cq_{\Delta 3}}+2^{-\Delta Cq_{▾3}}\right)]\;x\;100.$$

The distribution of transcript levels in control and mutant LCLs was calculated by normalization to that of pooled control cDNAs (reference sample) using the ∆ΔCq method (Livak & Schmittgen, 2001).

Statistical analysis was performed using GraphPad Prism software (version 5.02). The significance of the results was established using the F test.

### 2.7 Digital PCR

The dPCR experiments were performed on a QuantStudio 3D dPCR 20K platform according to the manufacturer's instructions (Applied Biosystem, Foster City, CA). To detect *BRCA2* Δ3 transcripts, we used a FAM‐labeled custom designed TaqMan assay (Applied Biosystems) specific for the exon 2–4 junction (5′‐CAAAGCAG‐GAAGGAATG‐3′). To detect ▼3 transcripts, we used a 2′‐chloro‐7′phenyl‐1,4‐dichloro‐6‐carboxy‐fluorescein labeled (VIC‐labeled) predesigned TaqMan assay (Applied Biosystems, Hs00609076) specific for the exon 3–4 junction (5‐AATTAGACTTAG‐GAAGGAATGTTCC‐3′). All relative quantification experiments were performed combining Δ3 and ▼3 assays in individual chips. dPCR chips were analyzed in the QuantStudio 3D Analysis Suit Cloud software v2.0 (Applied Biosystem, Foster City, CA), defining FAM as target. Default settings were used in all cases. After reviewing automatic assessment of the chip quality by the software, only green flag chips (data meet all quality thresholds, review of the analysis result not required) and yellow flag chips (data meet all quality thresholds, but manual inspection is recommended) were considered for further analyses. We used the target/total ratio, FAM/(FAM+VIC), calculated by the software as a proxy for *BRCA2* exon 3 exclusion rate. Different amounts of each sample were individually tested in 20K chips, but only data from the chip with the highest precision (according to the analysis software) was included in the expression analysis shown in Figure 3.

### 2.8 Genotyping and sample sets

Direct genotyping of *BRCA2* c.68‐7T > A was conducted as part of the Collaborative Oncological Gene‐environment Study (COGS) detailed elsewhere (Michailidou et al., 2013). This study included genotype results from breast cancer cases and controls participating in the Breast Cancer Association Consortium (BCAC; http:// bcac.ccge.medschl.cam.ac.uk/), and from the carriers of assumed pathogenic variants in BRCA genes, participating in the Consortium of Investigators of Modifiers of *BRCA1/2* (CIMBA; http:// cimba.ccge.medschl.cam.ac.uk/). The BCAC and CIMBA datasets are described in de la Hoya et al., (2016). Information on breast tumor estrogen receptor and grade status were available for 189 variant carrier cases from BCAC. Via the Evidence‐based Network for the Interpretation of Germline Mutant Alleles (ENIGMA; https://enigmaconsortium.org/) (Spurdle et al., 2012), we identified 16 families recruited through familial cancer clinics where at least one member tested positive for *BRCA2* c.68‐7T > A, and test results (negative or positive) were available from at least one relative. All study participants had been previously enrolled into national or regional studies under ethically approved protocols.

### 2.9 Statistical methods

The association of the *BRCA2* c.68‐7T > A variant with breast cancer risk was evaluated in BCAC using logistic regression models, as previously detailed (de la Hoya et al., 2016).

In addition, multifactorial likelihood analysis was conducted as detailed in the Supp. Text. In brief, odds for causality were calculated based on carrier frequency and ages at diagnosis/interview in cases and controls, as previously described (Goldgar et al., 2004).

Bayes scores for segregation were derived as previously described (Thompson, Easton, & Goldgar, 2003).

Pathology likelihood ratios (LRs) were applied as indicated in Spurdle et al., (2014). The segregation scores, pathology LRs and case‐control LRs are mutually independent and were combined to derive a combined odds for causality as described previously (Goldgar et al., 2004; Goldgar et al., 2008). Prior probability of pathogenicity was assigned based on predicted effect of the variant on splicing, as derived in Vallee et al., (2016). Variant classification was based on the IARC 5‐tier scheme (Plon et al., 2008).

### 2.10 Mitomycin C (MMC) growth inhibition test and statistical analyses

A total of 3 × 10^6^ cells/ml were seeded in triplicate in 25 ml flasks and grown for 72 hr in the absence or in the presence of MMC (Sigma‐Aldrich) at a final concentration of 170 ng/ml. Percentage of viable cells was determined using trypan blue dye exclusion assay, following the manufacture's instruction (Sigma‐Aldrich). Statistical differences in cell viability after exposure to MMC compared to controls were determined by two‐tailed Student *t*‐test using GraphPad Prism software.

## 3 RESULTS

### 3.1 Transcript analyses

#### 3.1.1 Confirmation of Δ3 transcripts increase in variant carriers

**Figure 1:**
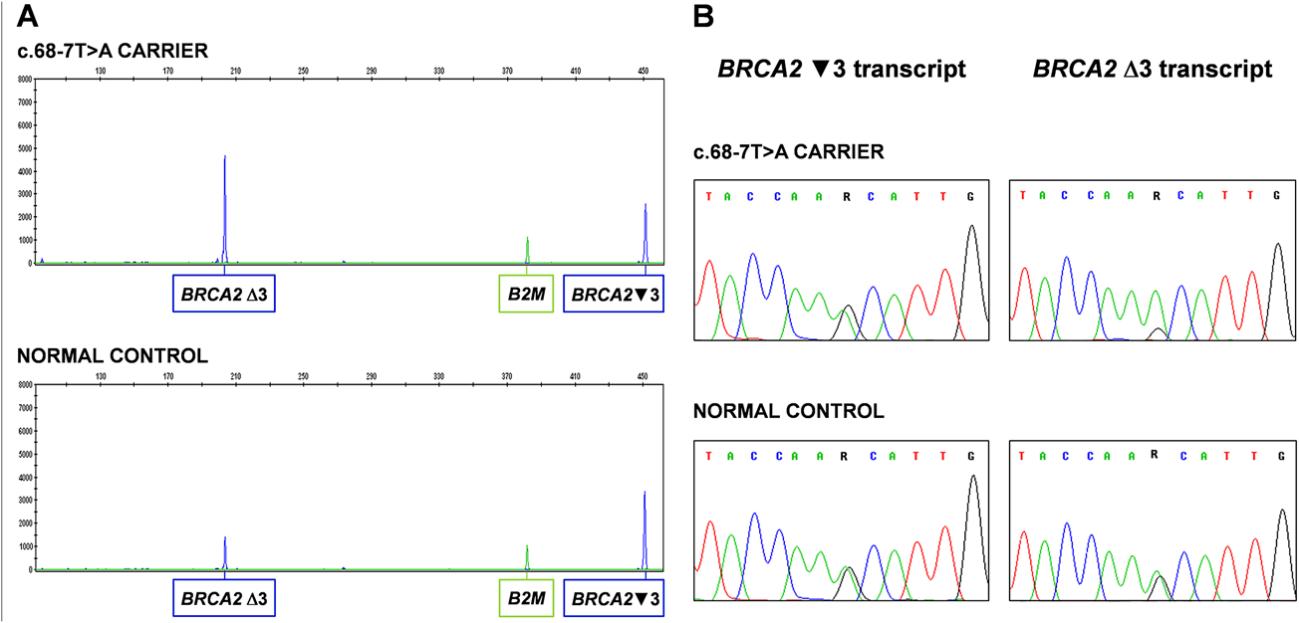
Evaluation of the effects of the *BRCA2* c.68‐7T > A variant at mRNA level. (A) Capillary electrophoresis analysis of *BRCA2* cDNA showing the relative increase of Δ3 transcript and decrease of ▼3 transcript in c.68‐7T > A carriers compared to normal controls *B2M* reference transcript. Since the PCR assays were performed under end‐point conditions, the results of these assays were not used to quantify the fold‐change of Δ3 versus ▼3 transcript ratio in cases compared to controls. (B) Assessment of allele‐specific expression of the ▼3 and ∆3 transcripts in c.68‐7T > A carriers and normal controls by analysis of the common c.‐26G > A variant. The sequencing of the RT‐PCR products obtained by selectively amplifying the ▼3 and ∆3 transcripts in separate reactions (left panels and right panels, respectively) shows that the variant allele, which is in linkage with the A allele of the common variant, retained the ability to synthesize the ▼3 transcript

The effect of the *BRCA2* c.68‐7T > A variant at the mRNA level was evaluated by fluorescently‐labeled end‐point RT‐PCR on cDNAs derived from six LCLs obtained from women carrying the investigated variant and from 12 nonvariant carrier females. The visual inspection of the CE outputs confirmed the increase of the Δ3 transcripts and the corresponding decrease of the ▼3 transcripts in variant carriers compared to controls (a representative example is shown in Fig. 1A), in agreement with previous studies (Houdayer et al., 2012; Jarhelle et al., 2016; Sanz et al., 2010; Thery et al., 2011; Vreeswijk et al., 2009).

The allelic‐specific expression of both the ▼3 and Δ3 transcripts was assessed by investigating the c.‐26G > A variant, verified to be in linkage with the c.68‐7T > A, in heterozygous samples (five controls and three cases). Each transcript was selectively amplified in separate reactions and sequenced. Even considering that transcript quantification by sequencing analysis is not entirely accurate, it was apparent that, while in normal samples the levels of the Δ3 transcripts originating from the two alleles were comparable, in carriers the contribution of the variant allele was higher than that of the wild‐type allele. In addition, it was also observed that in carriers the variant allele retained the ability to synthesize the ▼3 transcripts. A representative example is shown in Figure 1B.

#### 3.1.2 Quantitative mRNA analyses

**Figure 2:**
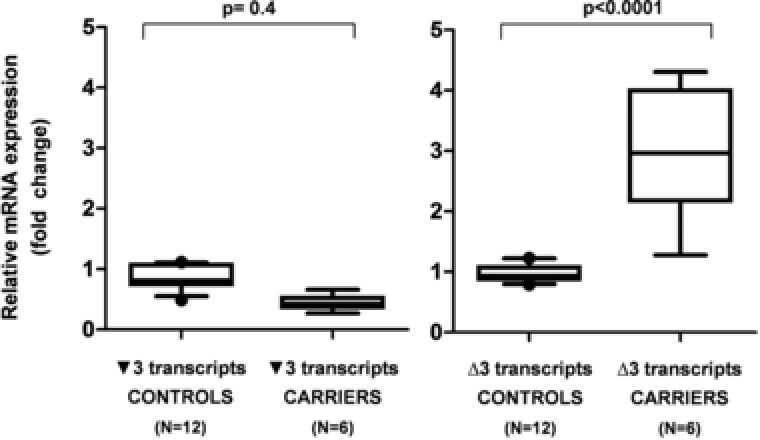
Relative expression of *BRCA2* ▼3 and ∆3 transcripts in six c.68‐7T > A carriers and 12 normal controls by quantitative PCR. The boxplots (displaying low, Q1, median, Q3, and high values) show qPCR levels of ▼3 and ∆3 transcripts in carriers and controls. Values are normalized to *GUSB* mRNA and expressed as fold difference relative to pooled control cDNAs using the ∆ΔCq method (see Materials and Methods). The analysis shows in carriers a statistically significant increase of the relative level of ∆3 transcripts compared to controls (2.98 vs. 0.97; *p* < 0.0001). Conversely, the decrease observed in the relative level of ▼3 transcripts (0,44 vs.0,86) is not statistically significant (*p* = 0.4)

To quantify the relative amount of *BRCA2* ▼3 and ∆3 transcripts in LCLs from both normal individuals (*n* = 12) and variant carriers (*n* = 6), a qPCR analysis was performed. The analysis showed a 3.1‐fold increase in the relative level of ∆3 transcripts (*p* < 10^−4^) in carriers (average 2.98; range 1.28–4.31) compared to controls (average 0.97; range 0.79–1.23) and a 0.5‐fold not statistically significant (*p* = 0.4) decrease in the relative level of ▼3 transcripts in carriers (average 0.44; range 0.27–0.66) compared to controls (average 0.86; range 0.49–1.11), (Fig. 2).

**Figure 3:**
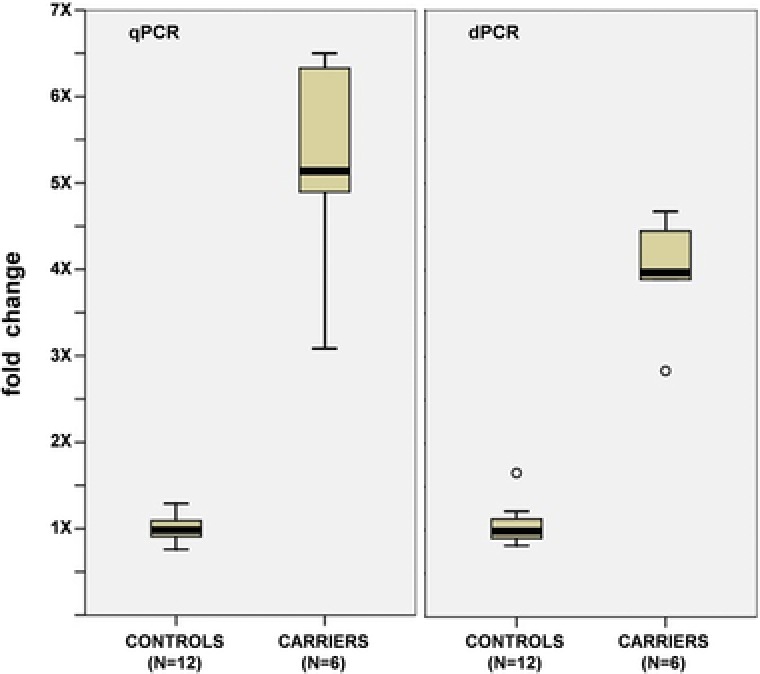
BRCA2 exon 3 exclusion rate in LCLs from *BRCA2* c.68‐7T > A carriers and controls. The boxplots (displaying low, Q1, median, Q3, and high values) show qPCR (left panel) and dPCR (right panel) measures of exclusion rate. The data is expressed as the fold‐increase relative to the average of 12 controls. Outliers (> 1.5 inter quartile range, IQR) are displayed as small circles. On average, a 5.2‐fold increase is observed in carriers according to qPCR data and a 4.2‐fold increase according to dPCR data (3.8‐fold increase if outliers are included in the analysis)

The relative quantification of ∆3 and ▼3 transcripts in each sample allowed us to compare the exon 3 exclusion rates (see methods) in carriers and controls and to obtain a quantitative score reflecting the magnitude of the splicing alteration induced by the variant. The exclusion rate in LCLs carrying the variant allele was 5.2‐fold higher than in normal LCLs (*p* = 3.9 × 10^−4^) (Fig. 3), with an average exclusion rate of 12.4% (range 6.3%–16.0%) in carriers and 2.4% (range 1.8%–3.4%) in controls (Supp. Figure S1).

Subsequently, an independent dPCR‐based quantitative analysis was performed to measure *BRCA2* exon 3 exclusion rates directly in the same sample set. After excluding two outliers, we found that the exclusion rate in LCLs carrying the variant allele (15.5%; range 14.4%–17.2%) was 4.2‐fold higher than in normal LCLs (3.7%; range 3.0%–4.5%; *p* < 10^−4^) (Fig. 3 and Supp. Figure S1).

### 3.2 Genetic analyses

*BRCA2* c.68‐7T > A was identified in 242/41,890 (0.58%) invasive breast cancer cases and 216/41,746 (0.52%) controls of reported European ancestry recruited through BCAC studies. Standard case‐control analysis adjusted for six principle components yielded an odds ratio (OR) of 1.03 (95% CI 0.86–1.24). However, some studies indicated that they had performed *BRCA1/2* mutation screening of cases and might have excluded cases with *BRCA1/2* VUS. This could have created a bias due to preferential exclusion of c.68‐7T > A carrier cases but not controls. However, the OR was similar after exclusion of four studies that performed such genetic testing, (OR 1.09; 95% CI 0.89–1.33). The odds for causality based on carrier frequency and ages at diagnosis/interview in these cases and controls was 9.44 × 10^−93^. There was also strong evidence against causality from segregation analysis (6.39 × 10^−9^) and breast tumor pathology (2.40 × 10^−14^). Considering all data together, and assigning prior probability of 0.34 based on splicing prediction, the posterior probability of pathogenicity was calculated to be 7.44 × 10^−115^ (see Supp. Text for further details).

### 3.3 Co‐occurrence of the c.68‐7T > A with pathogenic variants

Overall 15 female individuals from 13 apparently unrelated families with clear evidence of the c.68‐7T > A being in trans with a pathogenic variant in *BRCA2* were assessed. Thirteen individuals from 11 families were detected through the genotyping of the CIMBA sample set, one was reported via the ENIGMA consortium, and another one was ascertained at INT (Supp. Table S2). None of these cases was included in the RNA analyses described above.

### 3.4 Evaluation of the effect of the *BRCA2* c.68‐7T > A on cellular sensitivity to mitomycin C

Carriers of bi‐allelic *BRCA2* inactivating variants are affected with Fanconi Anemia (FA), complementation group D1. FA is characterized by congenital defects, including anatomical abnormalities, congenital disabilities and increased risk of cancer, most often acute myelogenous leukemia (Auerbach, 2009). In addition, the cells of FA patients exhibit hypersensitivity to DNA interstrand cross‐links (ICLs) caused by agents such as mitomycin C (MMC) (Godthelp et al., 2006). A breast cancer‐affected woman, with no clinical signs of FA, was found by segregation analysis to carry the truncating *BRCA2* c.5722_5723delCT variant in trans with the c.68‐7T > A variant (Supp. Table S2). To exclude an FA phenotype at the cellular level, we evaluated the sensitivity to MMC of an LCL derived from this patient. An LCL carrying one copy of the c.68‐7T > A, without an additional *BRCA2* pathogenic variant or VUS (*BRCA2* ^wt/c.68‐7T>A^), the MMC hypersensitive EUFA423 and Capan1 *BRCA2*‐null cell lines and an LCL from a normal donor (*BRCA2‐*proficient) were included in the assay as controls. The sensitivity to MMC was evaluated by comparing the viability of MMC‐treated cells with that of the untreated cells. As shown in Supp. Figure S2, EUFA423 (FA‐D1) and Capan1 cells showed a significant decrease of the cellular viability (*p* < 0.01) after exposure to MMC, while no differences were observed in LCLs from normal donor and carriers of *BRCA2* c.68‐7T > A, either in heterozygosity or in trans with the pathogenic variant.

## 4 DISCUSSION

In the present study, we analyzed the *BRCA2* c.68‐7T > A variant, located in the proximity of the acceptor site of exon 3, in order to establish its clinical relevance and association with breast cancer risk. In accordance with previous studies (Houdayer et al., 2012; Jarhelle et al., 2016; Sanz et al., 2010; Thery et al., 2011; Vreeswijk et al., 2009), we observed that this variant leads to a modest increased expression of the transcript lacking exon 3 (∆3) in carriers compared to controls. Moreover, we found that in LCLs of carriers of the variant the exon 3 exclusion rate (i.e., the relative amount of *BRCA2* ∆3 transcripts) was approximately 4‐ to 5‐fold higher than in LCLs of controls and the total amount of ▼3 transcripts in carriers was approximately 50% compared to controls. The latter finding would seem to contradict the observation that the variant allele maintains the ability to express a transcript coding for a normal (full‐length) protein. The apparent discrepancy may be explained comparing the overall expression of *BRCA2* transcripts in cases and controls. In fact, summing up in each sample the amount of ▼3 and Δ3 transcripts assessed by qPCR, and setting as 1 the average expression of *BRCA2* mRNA observed in our cohort, we observed notable inter‐individual variability (ranging from 0.43 to 1.50), with many control samples clustering above the average (Supp. Figure S3). Hence, it is very much possible that the strong reduction in the amount of ▼3 transcripts observed in carriers simply reflects random inter‐individual variability in *BRCA2* gene expression levels.

Although the above findings were confirmed using two complementary assays (qPCR and dPCR), it must be noted that the outcomes of transcript quantification analyses may be influenced by the nature of examined biological material. Therefore, the magnitude of changes in transcript ratio associated with the c.68‐7A > T should be verified also in samples other than LCLs.

The pathogenic implication of *BRCA2* exon 3 deletion has been long debated. Exon 3 is 249‐bp long and its deletion does not alter the open reading frame. In addition, the ∆3 isoform has been described as one of the major naturally occurring alternatively splicing events in *BRCA2* (Fackenthal et al., 2016). However, the predicted protein product is expected to be lacking important functional activities. In fact, this exon codes for BRCA2 amino acids 23 to 106, including the C‐terminal portion of a primary transactivating domain (PAR, amino‐acid residues 18–60) and an auxiliary transactivating domain (AAR, residues 60–105) (Milner, Ponder, Hughes‐Davies, Seltmann, & Kouzarides, 1997), whose activity may be regulated by phosphorylation (Milner, Fuks, Hughes‐Davies, & Kouzarides, 2000). Interestingly, the region spanning residues 21–39 mediates the interaction with PALB2, a nuclear protein that promotes the stable intranuclear localization and accumulation of BRCA2, making possible its DNA recombinational repair and checkpoint functions, eliciting tumor suppression (Oliver, Swift, Lord, Ashworth, & Pearl, 2009; Xia et al., 2006). Moreover, the PALB2‐binding site directly overlaps that of EMSY, another nuclear protein that has endogenous transcriptional repressor activity (Hughes‐Davies et al., 2003).

Several *BRCA2* alterations causing the complete loss of exon 3 and the exclusive synthesis of ∆3 transcripts have been ascertained, including c.316 + 5G > C (Bonnet et al., 2008), c.316 + 3delA and c.68‐925_316 + 2889del (Muller et al., 2011) and c.156_157insAlu, a variant reported as a founder Portuguese mutation (Peixoto et al., 2009).

The characterization of the above variants supports the hypothesis that the exclusive synthesis of the ∆3 transcripts from one allele has a pathogenic effect. On the contrary, the association with cancer risk of variants that, like the c.68‐7T > A, increase the relative amount of ∆3 isoforms but maintain the ability of transcribe a full‐length mRNA, is presently unclear. Indeed, the classification of the variants with incomplete effects at the transcript level represents an important and challenging question. According to current ENIGMA criteria, splicing variants leading to in‐frame deletions, but maintaining the ability to produce mRNA transcript(s) predicted to encode intact full‐length protein, cannot be assumed as pathogenic or likely pathogenic, even if targeting clinical relevant domains. Such alterations require further investigation to assess pathogenicity.

To address the issue, we performed a multifactorial‐likelihood analysis combining the odds for causality derived from a large case‐control study, using the datasets of BCAC, pathology likelihood based on breast tumor phenotype, and co‐segregation data from ENIGMA. Overall, the posterior probability of c.68‐7T > A being pathogenic was 7.44 × 10^−115^. This value is well below the threshold established by ENIGMA for a *BRCA1/2* variant to be classified as class 1, that is, not pathogenic (probability of pathogenicity < 0.001), when considered against characteristics of the average truncating pathogenic variant. In addition, the confidence interval of the odds ratio estimate (OR 1.09: 95%CI 0.89–1.33) excludes even moderate breast cancer risk (Hollestelle, Wasielewski, Martens, & Schutte, 2010).

Additional evidence of the non‐pathogenicity of c.68‐7T > A was provided by the observation of its occurrence in trans with a *BRCA2* pathogenic variant in 15 unrelated individuals, including 13 from 11 of 5,284 families recruited by CIMBA and genotyped for the variant. If c.68‐7T > A were pathogenic, the frequency of unrelated FA affected individuals among CIMBA *BRCA2* mutation carriers would be approximately 2.1 in 1,000, which is inconsistent with the frequency observed in the general population, that is, two to six in 1,000,000 (Bogliolo & Surralles, 2015). Finally, no evidence of hypersensitivity to DNA ICL agents, a characteristic of FA patients, was detected in an LCL derived from one of the individuals carrying a pathogenic variant in trans with the c.68‐7T > A. Together, these findings indicate that carriers of the *BRCA2* c.68‐7T > A variant should not be counseled to undergo the clinical interventions recommended to carriers of high risk BRCA gene variants.

While the present article was under review, a study was published claiming that the *BRCA2* c.68‐7T > A variant was associated with breast cancer (Møller & Hovig, 2017). This conclusion was based on the detection of the variant in 17 out of 714 (2.4%; 95%CI 1.4%–3.8%) Norwegian unrelated breast cancer kindreds, a frequency significantly higher (*p* < 0.0001) compared to the prevalence of the variant in a sample of the Norwegian population (3/1588 = 0.2%). Segregation data based on a single family was inconclusive (LR 0.36), and the estimate of prospective incidence rate in 24 variant carriers overlapped that for the general population. The authors concluded (assumedly based on their case‐control findings alone) that carriers of the *BRCA2* c.68‐7T > A variant have increased risk for breast cancer in families selected due to aggregation of breast cancer, and state in their discussion “…*carriers of the variant should be informed that they probably have a clinically actionable pathogenic variant and referred to health care accordingly*”. We believe that the conclusion of Moller and Hovig (2017) is unjustified, and disagree with their recommendation on clinical action. Our much larger study (sample size 59x for cases and 26x for controls) including individuals from multiple different countries provide no evidence for increased risk of breast cases in familial cases carrying this variant: the OR was 1.03 (95% CI 0.86–1.24) including all studies, and the risk estimate was nominally greater although not significantly different (OR 1.09, 95% CI 0.89–1.33) after excluding familial breast cancer cohorts.

The difference between the findings from our much larger case‐control study and that of Møller & Hovig, (2017) need for caution when utilizing case‐control data for clinical interpretation of rare variants, such that significant differences in frequency can nonetheless be unreliable due to random error and bias arising from small sample size, incomplete matching of cases and controls, and when considering familial cases, co‐occurrence of (other) risk‐related genetic factors as acknowledged by the authors themselves.

Different hypotheses, not necessarily mutually exclusive, can be proposed to explain the lack of pathogenicity of c.68‐7T > A despite it being spliceogenic. First, the reduction in full‐length *BRCA2* mRNAs in variant carriers compared to normal controls, which was not statistically significant, might not be enough to affect cellular tumor suppressor ability. Second, the ∆3 transcripts are predicted to lead to the synthesis of an unstable and nonfunctional protein product and, therefore, unlikely to interfere with the activity of the normal protein due to the loss of the PALB2 interaction domain, whose binding stabilizes the *BRCA2* protein (Xia et al., 2006). Assuming that in the examined samples, the overall *BRCA2* expression level from both alleles is similar, and that in carrier samples the accompanying normal alleles contribute on average an exclusion rate of approximately 3% as assessed by our quantitative analyses, we estimated, based on an average cumulative exclusion rate of both alleles in variant carriers of 13%, that the average exclusion rate (x) for the c.68‐7T > A allele is close to 23% [(x% + 3%)/2 = 13%.]. Therefore, the present study strongly suggests that *BRCA2* spliceogenic alleles demonstrating up to approximately 20% exon 3 exclusion rates are not associated with high or even moderate risk of cancer.

The classification of variants based on mRNA splicing data alone is problematic for spliceogenic variants that lead to equivocal or “leaky” transcript profiles. The quantitative in vitro transcript and genetic analyses conducted for *BRCA2* c.68‐7T > C provide important data to inform the threshold for ratio between functionally proficient and altered *BRCA2* isoforms compatible with normal cell function. These findings might facilitate the future classification of rare spliceogenic variants whose relevance for cancer risk cannot easily be ascertained through multifactorial likelihood analyses.

## CONFLICT OF INTEREST

The authors declare no conflict of interest.

## Supporting information

Supporting Information

Supporting Information

## References

[humu23411-bib-0001] Auerbach, A. D. (2009). Fanconi anemia and its diagnosis. Mutation Research, 668(1‐2), 4–10. https://doi.org/10.1016/j.mrfmmm.2009.01.013. 19622403 10.1016/j.mrfmmm.2009.01.013 PMC2742943

[humu23411-bib-0002] Bogliolo, M. , & Surrallés, J. (2015). Fanconi anemia: A model disease for studies on human genetics and advanced therapeutics. Current Opinion in Genetics & Development, 33, 32–40. https://doi.org/10.1016/j.gde.2015.07.002. 26254775 10.1016/j.gde.2015.07.002

[humu23411-bib-0003] Bonnet, C. , Krieger, S. , Vezain, M. , Rousselin, A. , Tournier, I. , Martins, A. , … Tosi, M. (2008). Screening BRCA1 and BRCA2 unclassified variants for splicing mutations using reverse transcription PCR on patient RNA and an ex vivo assay based on a splicing reporter minigene. Journal of Medical Genetics, 45, 438–446. https://doi.org/10.1136/jmg.2007.056895. 18424508 10.1136/jmg.2007.056895

[humu23411-bib-0004] Colombo, M. , De Vecchi, G. , Caleca, L. , Foglia, C. , Ripamonti, C. B. , Ficarazzi, F. , … Radice, P. (2013). Comparative in vitro and in silico analyses of variants in splicing regions of BRCA1 and BRCA2 genes and characterization of novel pathogenic mutations. PLOS One, 8(2), e57173 https://doi.org/10.1371/journal.pone.0057173. 23451180 10.1371/journal.pone.0057173 PMC3579815

[humu23411-bib-0005] de Brouwer, A. P. , van Bokhoven, H. , & Kremer, H. (2006). Comparison of 12 reference genes for normalization of gene expression levels in Epstein‐Barr virus‐transformed lymphoblastoid cell lines and fibroblasts. Molecular Diagnosis & Therapy, 10(3), 197–204. 16771605 10.1007/BF03256458

[humu23411-bib-0006] de la Hoya, M. , Soukarieh, O. , Lopez‐Perolio, I. , Vega, A. , Walker, L. C. , van Ierland, Y. , … Spurdle, A. B. (2016). Combined genetic and splicing analysis of BRCA1 c.[594‐2A>C; 641A>G] highlights the relevance of naturally occurring in‐frame transcripts for developing disease gene variant classification algorithms. Human Molecular Genetics, 25(11), 2256–2268. https://doi.org/10.1093/hmg/ddw094. 27008870 10.1093/hmg/ddw094 PMC5081057

[humu23411-bib-0007] Fackenthal, J. D. , Yoshimatsu, T. , Zhang, B. , de Garibay, G. R. , Colombo, M. , De Vecchi, G. , … de la Hoya, M. (2016). Naturally occurring BRCA2 alternative mRNA splicing events in clinically relevant samples. Journal of Medical Genetics, 53(8), 548–558. https://doi.org/10.1136/jmedgenet-2015-103570. 27060066 10.1136/jmedgenet-2015-103570

[humu23411-bib-0008] Feng, Z. , Scott, S. P. , Bussen, W. , Sharma, G. G. , Guo, G. , Pandita, T. K. , & Powell, S. N. (2011). Rad52 inactivation is synthetically lethal with BRCA2 deficiency. Proceedings of the National Academy of Sciences of the United States of America, 108(2), 686–691. https://doi.org/10.1073/pnas.1010959107. 21148102 10.1073/pnas.1010959107 PMC3021033

[humu23411-bib-0009] Godthelp, B. C. , van Buul, P. P. , Jaspers, N. G. , Elghalbzouri‐Maghrani, E. , van Duijn‐Goedhart, A. , Arwert, F. , … Zdzienicka, M. Z. (2006). Cellular characterization of cells from the Fanconi anemia complementation group, FA‐D1/BRCA2. Mutation Research, 601(1‐2), 191–201. https://doi.org/10.1016/j.mrfmmm.2006.07.003. 16920162 10.1016/j.mrfmmm.2006.07.003

[humu23411-bib-0010] Goggins, M. , Schutte, M. , Lu, J. , Moskaluk, C. A. , Weinstein, C. L. , Petersen, G. M. , … Kern, S. E. (1996). Germline BRCA2 gene mutations in patients with apparently sporadic pancreatic carcinomas. Cancer Research, 56(23), 5360–5364. 8968085

[humu23411-bib-0011] Goldgar, D. E. , Easton, D. F. , Byrnes, G. B. , Spurdle, A. B. , Iversen, E. S. , & Greenblatt, M. S. (2008). Genetic evidence and integration of various data sources for classifying uncertain variants into a single model. Human Mutation, 29, 1265–1272. https://doi.org/10.1002/humu.20897. 18951437 10.1002/humu.20897 PMC2936773

[humu23411-bib-0012] Goldgar, D. E. , Easton, D. F. , Deffenbaugh, A. M. , Monteiro, A. N. , Tavtigian, S. V. , & Couch, F. J. (2004). Integrated evaluation of DNA sequence variants of unknown clinical significance: Application to BRCA1 and BRCA2. The American Journal of Human Genetics, 75, 535–544. https://doi.org/10.1086/424388. 15290653 10.1086/424388 PMC1182042

[humu23411-bib-0013] Hollestelle, A. , Wasielewski, M. , Martens, J. W. , & Schutte, M. (2010). Discovering moderate‐risk breast cancer susceptibility genes. Current Opinion in Genetics & Development, 20(3), 268–276. https://doi.org/10.1016/j.gde.2010.02.009. 20346647 10.1016/j.gde.2010.02.009

[humu23411-bib-0014] Houdayer, C. , Caux‐Moncoutier, V. , Krieger, S. , Barrois, M. , Bonnet, F. , Bourdon, V. , … Stoppa‐Lyonnet, D. (2012). Guidelines for splicing analysis in molecular diagnosis derived from a set of 327 combined in silico/in vitro studies on BRCA1 and BRCA2 variants. Human Mutation, 33(8), 1228–1238. https://doi.org/10.1002/humu.22101. 22505045 10.1002/humu.22101

[humu23411-bib-0015] Howlett, N. G. , Taniguchi, T. , Olson, S. , Cox, B. , Waisfisz, Q. , De Die‐Smulders, C. , … D'Andrea, A. D. (2002). Biallelic inactivation of BRCA2 in Fanconi anemia. Science, 297(5581), 606–609. https://doi.org/10.1126/science.1073834. 12065746 10.1126/science.1073834

[humu23411-bib-0016] Hughes‐Davies, L. , Huntsman, D. , Ruas, M. , Fuks, F. , Bye, J. , Chin, S. F. , … Kouzarides, T. (2003). EMSY links the BRCA2 pathway to sporadic breast and ovarian cancer. Cell, 115, 523–535. 14651845 10.1016/s0092-8674(03)00930-9

[humu23411-bib-0017] Jarhelle, E. , Riise Stensland, H. M. , Maehle, L. , & Van Ghelue, M. (2016). Characterization of BRCA1 and BRCA2 variants found in a Norwegian breast or ovarian cancer cohort. Familial Cancer, 16(1), 1–16. https://doi.org/10.1007/s10689-016-9916-2. 10.1007/s10689-016-9916-2 27495310

[humu23411-bib-0018] Lek, M. , Karczewski, K. J. , Minikel, E. V. , Samocha, K. E. , Banks, E. , Fennell, T. , … MacArthur, D. G. (2016). Analysis of protein‐coding genetic variation in 60,706 humans. Nature, 536(7616), 285–291. https://doi.org/10.1038/nature19057. 27535533 10.1038/nature19057 PMC5018207

[humu23411-bib-0019] Livak, K. J. , & Schmittgen, T. D. (2001). Analysis of relative gene expression data using real‐time quantitative PCR and the 2(‐Delta Delta C(T)) Method. Methods (San Diego, California), 25(4), 402–408. https://doi.org/10.1006/meth.2001.1262. 10.1006/meth.2001.1262 11846609

[humu23411-bib-0020] Michailidou, K. , Hall, P. , Gonzalez‐Neira, A. , Ghoussaini, M. , Dennis, J. , Milne, R. L. , … Easton, D. F. (2013). Large‐scale genotyping identifies 41 new loci associated with breast cancer risk. Nature Genetics, 45(4), 353–362. https://doi.org/10.1038/ng.2563. 23535729 10.1038/ng.2563 PMC3771688

[humu23411-bib-0021] Milner, J. , Fuks, F. , Hughes‐Davies, L. , & Kouzarides, T. (2000). The BRCA2 activation domain associates with and is phosphorylated by a cellular protein kinase. Oncogene, 19(38), 4441–4445. https://doi.org/10.1038/sj.onc.1203793. 10980621 10.1038/sj.onc.1203793

[humu23411-bib-0022] Milner, J. , Ponder, B. , Hughes‐Davies, L. , Seltmann, M. , & Kouzarides, T. (1997). Transcriptional activation functions in BRCA2. Nature, 386, 772–773. https://doi.org/10.1038/386772a0. 9126734 10.1038/386772a0

[humu23411-bib-0023] Møller, P. , & Hovig, E. (2017). The BRCA2 variant c.68‐7 T>A is associated with breast cancer. Hereditary Cancer in Clinical Practice, 15, 20 https://doi.org/10.1186/s13053-017-0080-y. 29158857 10.1186/s13053-017-0080-y PMC5683587

[humu23411-bib-0024] Muller, D. , Rouleau, E. , Schultz, I. , Caputo, S. , Lefol, C. , Bieche, I. , … Abecassis, J. (2011). An entire exon 3 germ‐line rearrangement in the BRCA2 gene: Pathogenic relevance of exon 3 deletion in breast cancer predisposition. BMC Medical Genetics, 12, 121–112. https://doi.org/10.1186/1471-2350-12-121. 21939546 10.1186/1471-2350-12-121 PMC3198910

[humu23411-bib-0025] Oliver, A. W. , Swift, S. , Lord, C. J. , Ashworth, A. , & Pearl, L. H. (2009). Structural basis for recruitment of BRCA2 by PALB2. EMBO Reports, 10(9), 990–996. https://doi.org/10.1038/embor.2009.126. 19609323 10.1038/embor.2009.126 PMC2750052

[humu23411-bib-0026] Peixoto, A. , Santos, C. , Rocha, P. , Pinheiro, M. , Principe, S. , Pereira, D. , … Teixeira, M. R. (2009). The c.156_157insAlu BRCA2 rearrangement accounts for more than one‐fourth of deleterious BRCA mutations in northern/central Portugal. Breast Cancer Research and Treatment, 114(1), 31–38.https://doi.org/10.1007/s10549-008-9978-4. 18363094 10.1007/s10549-008-9978-4

[humu23411-bib-0027] Plon, S. E. , Eccles, D. M. , Easton, D. , Foulkes, W. D. , Genuardi, M. , Greenblatt, M. S. , … Tavtigian, S. V. (2008). Sequence variant classification and reporting: Recommendations for improving the interpretation of cancer susceptibility genetic test results. Human Mutation, 29(11), 1282–1291. https://doi.org/10.1002/humu.20880. 18951446 10.1002/humu.20880 PMC3075918

[humu23411-bib-0028] Santos, C. , Peixoto, A. , Rocha, P. , Pinto, P. , Bizarro, S. , Pinheiro, M. , … Teixeira, M. R. (2014). Pathogenicity evaluation of BRCA1 and BRCA2 unclassified variants identified in Portuguese breast/ovarian cancer families. The Journal of Molecular Diagnostics, 16(3), 324–334. https://doi.org/10.1016/j.jmoldx.2014.01.005. 24607278 10.1016/j.jmoldx.2014.01.005

[humu23411-bib-0029] Sanz, D. J. , Acedo, A. , Infante, M. , Duran, M. , Perez‐Cabornero, L. , Esteban‐Cardenosa, E. , … Velasco, E. A. (2010). A high proportion of DNA variants of BRCA1 and BRCA2 is associated with aberrant splicing in breast/ovarian cancer patients. Clinical Cancer Research, 16, 1957–1967. https://doi.org/10.1158/1078-0432.CCR-09-2564. 20215541 10.1158/1078-0432.CCR-09-2564

[humu23411-bib-0030] Spurdle, A. B. , Couch, F. J. , Parsons, M. T. , McGuffog, L. , Barrowdale, D. , Bolla, M. K. , … Goldgar, D. E. (2014). Refined histopathological predictors of BRCA1 and BRCA2 mutation status: A large‐scale analysis of breast cancer characteristics from the BCAC, CIMBA, and ENIGMA consortia. Breast Cancer Research, 16(6), 3419–0474. https://doi.org/10.1186/s13058-014-0474-y. 25857409 10.1186/s13058-014-0474-y PMC4352262

[humu23411-bib-0031] Spurdle, A. B. , Healey, S. , Devereau, A. , Hogervorst, F. B. , Monteiro, A. N. , Nathanson, K. L. , … Goldgar, D. E. (2012). ENIGMA–evidence‐based network for the interpretation of germline mutant alleles: An international initiative to evaluate risk and clinical significance associated with sequence variation in BRCA1 and BRCA2 genes. Human Mutation, 33(1), 2–7. https://doi.org/10.1002/humu.21628. 21990146 10.1002/humu.21628 PMC3240687

[humu23411-bib-0032] Thery, J. C. , Krieger, S. , Gaildrat, P. , Revillion, F. , Buisine, M. P. , Killian, A. , … Tosi, M. (2011). Contribution of bioinformatics predictions and functional splicing assays to the interpretation of unclassified variants of the BRCA genes. European Journal of Human Genetics, 19(10), 1052–1058. https://doi.org/10.1038/ejhg.2011.100. 21673748 10.1038/ejhg.2011.100 PMC3190263

[humu23411-bib-0033] Thompson, D. , Easton, D. F. , & Goldgar, D. E. (2003). A full‐likelihood method for the evaluation of causality of sequence variants from family data. The American Journal of Human Genetics, 73, 652–655. https://doi.org/10.1086/378100. 12900794 10.1086/378100 PMC1180690

[humu23411-bib-0034] Vallee, M. P. , Di Sera, T. L. , Nix, D. A. , Paquette, A. M. , Parsons, M. T. , Bell, R. , … Tavtigian, S. V. (2016). Adding In Silico Assessment of Potential Splice Aberration to the Integrated Evaluation of BRCA Gene Unclassified Variants. Human Mutation, 37(7), 627–639. https://doi.org/10.1002/humu.22973. 26913838 10.1002/humu.22973 PMC4907813

[humu23411-bib-0035] Vreeswijk, M. P. , Kraan, J. N. , van der Klift, H. M. , Vink, G. R. , Cornelisse, C. J. , Wijnen, J. T. , … Devilee, P. (2009). Intronic variants in BRCA1 and BRCA2 that affect RNA splicing can be reliably selected by splice‐site prediction programs. Human Mutation, 30, 107–114. https://doi.org/10.1002/humu.20811. 18693280 10.1002/humu.20811

[humu23411-bib-0036] Xia, B. , Sheng, Q. , Nakanishi, K. , Ohashi, A. , Wu, J. , Christ, N. , … Livingston, D. M. (2006). Control of BRCA2 cellular and clinical functions by a nuclear partner, PALB2. Molecular Cell, 22(6), 719–729. https://doi.org/10.1016/j.molcel.2006.05.022. 16793542 10.1016/j.molcel.2006.05.022

