# Supplementary material for: The BRCA2 c.68‐7T > A variant is not pathogenic: A model for clinical calibration of spliceogenicity
Source: Hum Mutat. 2018 Apr 6;39(5):729–41. doi: 10.1002/humu.23411 (PMC5947288; doi:10.1002/humu.23411)
Supplement: Supplementary file 1 — Supporting Information [file HUMU-39-729-s001.docx]

**FUNDING**

The research was supported by Italian Association for Cancer Research [AIRC; grant N°15547.to P.R.], Spanish Instituto de Salud Carlos III funding, an initiative of the Spanish Ministry of Economy and Innovation partially supported by European Regional Development FEDER Funds [PI15/00059 to M.d.H.], and the Cancer Council Queensland [APP1086286 to A.B.S.]; the NHMRC Senior Research Fellowship Scheme [ID 1061779 to A.B.S.]; NHMRC Project grant scheme [ID 1010719 to A.B.S.].

ENIGMA Collaborators

Institutional grants “5x1000” Istituto Oncologico Veneto IRCCS to M.M. and IRCCS AOU San Martino – IST to L.V.

BCAC Studies

The Australian Breast Cancer Family Study (**ABCFS**) was supported by grant UM1 CA164920 from the National Cancer Institute (USA). The content of this manuscript does not necessarily reflect the views or policies of the National Cancer Institute or any of the collaborating centers in the Breast Cancer Family Registry (BCFR), nor does mention of trade names, commercial products, or organizations imply endorsement by the USA Government or the BCFR. The ABCFS was also supported by the National Health and Medical Research Council of Australia, the New South Wales Cancer Council, the Victorian Health Promotion Foundation (Australia) and the Victorian Breast Cancer Research Consortium. J.L.H. is a National Health and Medical Research Council (NHMRC) Senior Principal Research Fellow. M.C.S. is a NHMRC Senior Research Fellow. The **ABCS** study was supported by the Dutch Cancer Society [grants NKI 2007-3839; 2009 4363]. The work of the **BBCC** was partly funded by ELAN-Fond of the University Hospital of Erlangen. The **BBCS** is funded by Cancer Research UK and Breast Cancer Now and acknowledges NHS funding to the NIHR Biomedical Research Centre, and the National Cancer Research Network (NCRN). The **BCAC** is funded by Cancer Research UK [C1287/A16563, C1287/A10118], the European Union's Horizon 2020 Research and Innovation Programme (grant numbers 634935 and 633784 for BRIDGES and B-CAST respectively), and by the European Community´s Seventh Framework Programme under grant agreement number 223175 (grant number HEALTH-F2-2009-223175) (COGS). ES (**BIGGS**) is supported by NIHR Comprehensive Biomedical Research Centre, Guy's & St. Thomas' NHS Foundation Trust in partnership with King's College London, United Kingdom. IT is supported by the Oxford Biomedical Research Centre. BRCA-gene mutations and breast cancer in South African women (**BMBSA**) was supported by grants from the Cancer Association of South Africa (CANSA) to Elizabeth J. van Rensburg. The **BSUCH** study was supported by the Dietmar-Hopp Foundation, the Helmholtz Society and the German Cancer Research Center (DKFZ). The **CECILE** study was supported by Fondation de France, Institut National du Cancer (INCa), Ligue Nationale contre le Cancer, Agence Nationale de Sécurité Sanitaire, de l'Alimentation, de l'Environnement et du Travail (ANSES), Agence Nationale de la Recherche (ANR). The **CGPS** was supported by the Chief Physician Johan Boserup and Lise Boserup Fund, the Danish Medical Research Council, and Herlev and Gentofte Hospital. The **CNIO-BCS** was supported by the Instituto de Salud Carlos III, the Red Temática de Investigación Cooperativa en Cáncer and grants from the Asociación Española Contra el Cáncer and the Fondo de Investigación Sanitario (PI11/00923 and PI12/00070). The **CTS** was initially supported by the California Breast Cancer Act of 1993 and the California Breast Cancer Research Fund (contract 97-10500) and is currently funded through the National Institutes of Health (R01 CA77398, UM1 CA164917, and U01 CA199277). Collection of cancer incidence data was supported by the California Department of Public Health as part of the statewide cancer reporting program mandated by California Health and Safety Code Section 103885. HAC receives support from the Lon V Smith Foundation (LVS39420). The **ESTHER** study was supported by a grant from the Baden Württemberg Ministry of Science, Research and Arts. Additional cases were recruited in the context of the VERDI study, which was supported by a grant from the German Cancer Aid (Deutsche Krebshilfe). The German Consortium of Hereditary Breast and Ovarian Cancer (**GC-HBOC**) is supported by the German Cancer Aid (grant no 110837, coordinator: Rita K. Schmutzler). The **GENICA** was funded by the Federal Ministry of Education and Research (BMBF) Germany grants 01KW9975/5, 01KW9976/8, 01KW9977/0 and 01KW0114, the Robert Bosch Foundation, Stuttgart, Deutsches Krebsforschungszentrum (DKFZ), Heidelberg, the Institute for Prevention and Occupational Medicine of the German Social Accident Insurance, Institute of the Ruhr University Bochum (IPA), Bochum, as well as the Department of Internal Medicine, Evangelische Kliniken Bonn gGmbH, Johanniter Krankenhaus, Bonn, Germany. The **HEBCS** was financially supported by the Helsinki University Central Hospital Research Fund, Academy of Finland (266528), the Finnish Cancer Society, The Nordic Cancer Union and the Sigrid Juselius Foundation. The **HMBCS** was supported by a grant from the Friends of Hannover Medical School and by the Rudolf Bartling Foundation. Financial support for **KARBAC** was provided through the regional agreement on medical training and clinical research (ALF) between Stockholm County Council and Karolinska Institutet, the Swedish Cancer Society, The Gustav V Jubilee foundation and Bert von Kantzows foundation. The **KBCP** was financially supported by the special Government Funding (EVO) of Kuopio University Hospital grants, Cancer Fund of North Savo, the Finnish Cancer Organizations, and by the strategic funding of the University of Eastern Finland. **kConFab** is supported by a grant from the National Breast Cancer Foundation, and previously by the National Health and Medical Research Council (NHMRC), the Queensland Cancer Fund, the Cancer Councils of New South Wales, Victoria, Tasmania and South Australia, and the Cancer Foundation of Western Australia. G.C.T. is supported by the NHMRC. The **LMBC** is supported by the 'Stichting tegen Kanker'. Diether Lambrechts is supported by the FWO. The **MARIE** study was supported by the Deutsche Krebshilfe e.V. [70-2892-BR I, 106332, 108253, 108419, 110826, 110828], the Hamburg Cancer Society, the German Cancer Research Center (DKFZ) and the Federal Ministry of Education and Research (BMBF) Germany [01KH0402]. The **MBCSG** is supported by grants from the Italian Association for Cancer Research (AIRC) and by funds from the Italian citizens who allocated the 5/1000 share of their tax payment in support of the Fondazione IRCCS Istituto Nazionale Tumori, according to Italian laws (INT-Institutional strategic projects “5x1000” ). The **MCBCS** was supported by the NIH grants CA192393, CA116167, CA176785 an NIH Specialized Program of Research Excellence (SPORE) in Breast Cancer [CA116201], and the Breast Cancer Research Foundation and a generous gift from the David F. and Margaret T. Grohne Family Foundation. **MCCS** cohort recruitment was funded by VicHealth and Cancer Council Victoria. The MCCS was further supported by Australian NHMRC grants 209057, 251553 and 504711 and by infrastructure provided by Cancer Council Victoria. Cases and their vital status were ascertained through the Victorian Cancer Registry (VCR) and the Australian Institute of Health and Welfare (AIHW), including the National Death Index and the Australian Cancer Database. The **MEC** was support by NIH grants CA63464, CA54281, CA098758, CA132839 and CA164973. The **MYBRCA** is funded by research grants from the Malaysian Ministry of Higher Education (UM.C/HlR/MOHE/06) and Cancer Research Malaysia. MYMAMMO is supported by research grants from Yayasan Sime Darby LPGA Tournament and Malaysian Ministry of Higher Education (RP046B-15HTM). The **NBHS** was supported by NIH grant R01CA100374. Biological sample preparation was conducted the Survey and Biospecimen Shared Resource, which is supported by P30 CA68485. The **OBCS** was supported by research grants from the Finnish Cancer Foundation, the Academy of Finland (grant number 250083, 122715 and Center of Excellence grant number 251314), the Finnish Cancer Foundation, the Sigrid Juselius Foundation, the University of Oulu, the University of Oulu Support Foundation and the special Governmental EVO funds for Oulu University Hospital-based research activities. The Ontario Familial Breast Cancer Registry (**OFBCR**) was supported by grant UM1 CA164920 from the National Cancer Institute (USA). The content of this manuscript does not necessarily reflect the views or policies of the National Cancer Institute or any of the collaborating centers in the Breast Cancer Family Registry (BCFR), nor does mention of trade names, commercial products, or organizations imply endorsement by the USA Government or the BCFR. The **ORIGO** study was supported by the Dutch Cancer Society (RUL 1997-1505) and the Biobanking and Biomolecular Resources Research Infrastructure (BBMRI-NL CP16). The **PBCS** was funded by Intramural Research Funds of the National Cancer Institute, Department of Health and Human Services, USA. The **pKARMA** study was supported by Märit and Hans Rausings Initiative Against Breast Cancer. The **RBCS** was funded by the Dutch Cancer Society (DDHK 2004-3124, DDHK 2009-4318). The **SASBAC** study was supported by funding from the Agency for Science, Technology and Research of Singapore (A*STAR), the US National Institute of Health (NIH) and the Susan G. Komen Breast Cancer Foundation. The **SBCS** was supported by Sheffield Experimental Cancer Medicine Centre and Breast Cancer Now. The **SEARCH** is funded by a programme grant from Cancer Research UK [C490/A10124] and supported by the UK National Institute for Health Research Biomedical Research Centre at the University of Cambridge. The **SGBCC** is funded by the NUS start-up Grant, National University Cancer Institute Singapore (NCIS) Centre Grant and the NMRC Clinician Scientist Award. Additional controls were recruited by the Singapore Consortium of Cohort Studies-Multi-ethnic cohort (SCCS-MEC), which was funded by the Biomedical Research Council, grant number: 05/1/21/19/425. The **SZBCS** was supported by Grant PBZ_KBN_122/P05/2004. The **TNBCC** was supported by: a Specialized Program of Research Excellence (SPORE) in Breast Cancer (CA116201), a grant from the Breast Cancer Research Foundation, a generous gift from the David F. and Margaret T. Grohne Family Foundation; the Stefanie Spielman Breast Cancer fund and the OSU Comprehensive Cancer Center; the Hellenic Cooperative Oncology Group research grant (HR R_BG/04) and the Greek General Secretary for Research and Technology (GSRT) Program, Research Excellence II, the European Union (European Social Fund – ESF), and Greek national funds through the Operational Program "Education and Lifelong Learning" of the National Strategic Reference Framework (NSRF) - ARISTEIA. The **UKBGS** is funded by Breast Cancer Now and the Institute of Cancer Research (ICR), London. ICR acknowledges NHS funding to the NIHR Biomedical Research Centre.

CIMBA Studies

The **CIMBA** data management and data analysis were supported by Cancer Research – UK grants C12292/A20861, C12292/A11174. The **EMBRACE** is supported by Cancer Research UK Grants C1287/A10118, C1287/A11990 and C1287/A23382.The **HEBON** study is supported by the Dutch Cancer Society grants NKI1998-1854, NKI2004-3088, NKI2007-3756, the Netherlands Organisation of Scientific Research grant NWO 91109024, the Pink Ribbon grants 110005 and 2014-187.WO76, the BBMRI grant NWO 184.021.007/CP46 and the Transcan grant JTC 2012 Cancer 12-054. The **MSKCC** is supported by grants from the Breast Cancer Research Foundation, the Robert and Kate Niehaus Clinical Cancer Genetics Initiative, and the Andrew Sabin Research Fund.
