# Supplementary material for: The BRCA2 c.68‐7T > A variant is not pathogenic: A model for clinical calibration of spliceogenicity
Source: Hum Mutat. 2018 Apr 6;39(5):729–41. doi: 10.1002/humu.23411 (PMC5947288; doi:10.1002/humu.23411)
Supplement: Supplementary file 2 — Supporting Information [file HUMU-39-729-s002.docx]

**Colombo et al: The** ***BRCA2* c.68-7T>A variant is not pathogenic: a model for clinical calibration of spliceogenicity**

**Supplementary Text**

**Description of multifactorial likelihood analysis**

All multifactorial likelihood components compare the probability of observing the data assuming that the variant is pathogenic at the same level as the “average” truncating mutation compared to the probability assuming that the variant under study has no effect on risk. The information included in the multifactorial likelihood analysis for the *BRCA2* c.68-7T>A variant is described below.

**Prior probability**

The Prior Probability of 0.34 was assigned based on previous clinical calibration of splicing predictions of damage to the acceptor site (<http://priors.hci.utah.edu/PRIORS/>). The MaxEntScan prediction score changed from 6.10 (Z-score -0.77) for the wild-type sequence to 4.64 (Z-score 1.37) for the variant sequence, and is considered to be a moderate change by the criteria presented by Vallee et al (Vallee et al., 2016).

**Component Likelihoods**

*Case-control likelihood ratio:*

The odds for causality based on carrier frequency and ages at diagnosis (cases) or interview (controls) was 9.44 x 10^-93^, using the method described in de la Hoya et al (de la Hoya et al., 2016). For the Case Control Likelihood Ratio (LR) we first calculate the probability that a random case in the population carries the variant which is a function of the population allele frequency and the genotype relative risk conferred by the variant. This is divided by the probability that a random control in the population carries the variant, which is simply the carrier frequency allele frequency. This gives the LR that a variant is found in a case vs control. Next we calculate the posterior probability that the variant is found in a case in the sample using the prior probability (proportion of cases in the total sample) using Bayes rule. The probability that the variant is found in a control is just 1- P(found in case). These probabilities are multiplied across all variant carriers in the data set to get the likelihood of the data under the hypothesis that the variant is pathogenic. If the variant is not related to risk, the distribution of the number of variants found in in cases versus controls is simply a binomial distribution with probability equal to the proportion of cases in the total sample. The ratio of these two likelihoods is the case-control LR. The distribution of cases and controls in age-groups used to assign probabilities are denoted in the table below.

| **Age range**  (Diagnosis for cases; Interview for controls) | **N Cases** | **N Controls** |
| --- | --- | --- |
| 20-30) | 21 | 25 |
| 30-40) | 44 | 18 |
| 40-50) | 66 | 39 |
| 50-60) | 63 | 67 |
| 60-70) | 44 | 48 |
| 70-80) | 0 | 18 |
| 80+ | 0 | 0 |
| Unknown | 4 | 0 |

*Bayes score from segregation analysis:*

The Bayes score from segregation analysis including information from 16 informative families was 6.39 x 10^-9^. Bayes scores were calculated following the method of Thompson et al (Thompson et al., 2003), where the LR for each family is derived by comparing the likelihood of affected individuals sharing the variant with that under the null hypothesis (that the variant is non-pathogenic, in which case the variant will segregate randomly within a pedigree). The likelihood is a function of an assumed penetrance function incorporating the risk of disease for each genotype at the disease locus (which may be age- and/or sex-specific) and the frequency of the disease allele (usually assumed to be very rare). These penetrance parameters are typically assumed to be those estimated from (hopefully) large studies of families segregating known ‘‘high-risk’’ pathogenic mutations. LRs for individual families are combined by multiplication.

*Pathology likelihood ratio:*

The combined LR based on pathology information was 2.40 x 10^-14^, using information on ER and/or grade breast tumor status that was available for 189 variant carriers denoted as of European descent. The table below summarises pathology features of variant carriers, and the likelihoods applied, as per the decision flowchart in Spurdle et al (Spurdle et al., 2014).

|  |  | Grade | | | | | | | |
| --- | --- | --- | --- | --- | --- | --- | --- | --- | --- |
|  |  | 1 | | 2 | | 3 | | Unknown | |
|  | Age | <50 | ≥50 | <50 | ≥50 | <50 | ≥50 | <50 | ≥50 |
| ER | Positive | 9 | 21 | 9 | 52 | 2 | 23 | 9 | 12 |
|  | *LR calculation* | *0.37^9^* | *0.32^21^* | *1.07^9^* | *0.89^52^* | *1.77^2^* | *1.76^23^* | *1.15^9^* | *0.90^12^* |
|  | Negative | 0 | 1 | 3 | 6 | 7 | 12 | 3 | 4 |
|  | *LR calculation* | *N/A*  *(0.51)* | *0.86* | *0.49^3^* | *0.89^6^* | *0.69^7^* | *1.54^12^* | *0.66^3^* | *1.18^4^* |
|  | Unknown | 2 | 4 | 2 | 4 | 1 | 3 |  |  |
|  | *LR calculation* | *0.33^2^* | *0.35^4^* | *0.88^2^* | *0.81^4^* | *1.08* | *1.52^3^* |  |  |

LRs based on observations for each tumour pathology group were combined by multiplication eg 0.37^9^ x 0.32^21^ x 1.07^9^ x ….

*Combined odds for causality based on component likelihoods:*

The Odds for Causality is calculated as the product of the individual statistically independent components, namely (LR Case-control [9.44 x 10^-93^] x LR Segregation [6.39 x 10^-9^] x LR Pathology [2.40 x 10^-14^] = 1.45 x 10^-114^.

**Posterior probability**

The posterior probability of pathogenicity was calculated by using Bayes rule to combine the prior probability (based on clinically calibrated sequence information), and the combined odds for causality derived from component likelihood ratios (LRs). The calculation (and figures used for *BRCA2* c.68-7T>A) are shown below.

The Posterior Probability = Posterior Odds/(Posterior Odds+1), where the Posterior Odds = Prior Probability x Combined odds for causality x (1/1-prior probability).

| Variant | Prior | Segregation LR | Pathology LR | Case-Control LR | Combined Odds for causality | Posterior Odds | Posterior probability |
| --- | --- | --- | --- | --- | --- | --- | --- |
| *BRCA2*  c.68-7T>A | 0.34 | 6.38501E-09 | 2.39644E-14 | 9.43756E-93 | 1.4441E-114 | 7.4391E-115 | 7.4391E-115 |

**REFERENCES**

de la Hoya M, Soukarieh O, Lopez-Perolio I, Vega A, Walker LC, van IY, Baralle D, Santamarina M, Lattimore V, Wijnen J, Whiley P, Blanco A, Raponi M, Hauke J, Wappenschmidt B, Becker A, Hansen TV, Behar R, Investigators K, Niederacher D, Arnold N, Dworniczak B, Steinemann D, Faust U, Rubinstein W, Hulick PJ, Houdayer C, Caputo SM, Castera L, Pesaran T, Chao E, Brewer C, Southey MC, van Asperen CJ, Singer CF, Sullivan J, Poplawski N, Mai P, Peto J, Johnson N, Burwinkel B, Surowy H, Bojesen SE, Flyger H, Lindblom A, Margolin S, Chang-Claude J, Rudolph A, Radice P, Galastri L, Olson JE, Hallberg E, Giles GG, Milne RL, Andrulis IL, Glendon G, Hall P, Czene K, Blows F, Shah M, Wang Q, Dennis J, Michailidou K, McGuffog L, Bolla MK, Antoniou AC, Easton DF, Couch FJ, Tavtigian S, Vreeswijk MP, Parsons M, Meeks HD, Martins A, Goldgar DE, Spurdle AB. 2016. Combined genetic and splicing analysis of BRCA1 c.[594-2A>C; 641A>G] highlights the relevance of naturally occurring in-frame transcripts for developing disease gene variant classification algorithms. Hum Mol Genet 25(11):2256-2268.

[Spurdle AB](https://www.ncbi.nlm.nih.gov/pubmed/?term=Spurdle%20AB%5BAuthor%5D&cauthor=true&cauthor_uid=25857409), [Couch FJ](https://www.ncbi.nlm.nih.gov/pubmed/?term=Couch%20FJ%5BAuthor%5D&cauthor=true&cauthor_uid=25857409), [Parsons MT](https://www.ncbi.nlm.nih.gov/pubmed/?term=Parsons%20MT%5BAuthor%5D&cauthor=true&cauthor_uid=25857409), [McGuffog L](https://www.ncbi.nlm.nih.gov/pubmed/?term=McGuffog%20L%5BAuthor%5D&cauthor=true&cauthor_uid=25857409), [Barrowdale D](https://www.ncbi.nlm.nih.gov/pubmed/?term=Barrowdale%20D%5BAuthor%5D&cauthor=true&cauthor_uid=25857409), [Bolla MK](https://www.ncbi.nlm.nih.gov/pubmed/?term=Bolla%20MK%5BAuthor%5D&cauthor=true&cauthor_uid=25857409), [Wang Q](https://www.ncbi.nlm.nih.gov/pubmed/?term=Wang%20Q%5BAuthor%5D&cauthor=true&cauthor_uid=25857409), [Healey S](https://www.ncbi.nlm.nih.gov/pubmed/?term=Healey%20S%5BAuthor%5D&cauthor=true&cauthor_uid=25857409), [Schmutzler R](https://www.ncbi.nlm.nih.gov/pubmed/?term=Schmutzler%20R%5BAuthor%5D&cauthor=true&cauthor_uid=25857409), [Wappenschmidt B](https://www.ncbi.nlm.nih.gov/pubmed/?term=Wappenschmidt%20B%5BAuthor%5D&cauthor=true&cauthor_uid=25857409), [Rhiem K](https://www.ncbi.nlm.nih.gov/pubmed/?term=Rhiem%20K%5BAuthor%5D&cauthor=true&cauthor_uid=25857409), [Hahnen E](https://www.ncbi.nlm.nih.gov/pubmed/?term=Hahnen%20E%5BAuthor%5D&cauthor=true&cauthor_uid=25857409), [Engel C](https://www.ncbi.nlm.nih.gov/pubmed/?term=Engel%20C%5BAuthor%5D&cauthor=true&cauthor_uid=25857409), [Meindl A](https://www.ncbi.nlm.nih.gov/pubmed/?term=Meindl%20A%5BAuthor%5D&cauthor=true&cauthor_uid=25857409), [Ditsch N](https://www.ncbi.nlm.nih.gov/pubmed/?term=Ditsch%20N%5BAuthor%5D&cauthor=true&cauthor_uid=25857409), [Arnold N](https://www.ncbi.nlm.nih.gov/pubmed/?term=Arnold%20N%5BAuthor%5D&cauthor=true&cauthor_uid=25857409), [Plendl H](https://www.ncbi.nlm.nih.gov/pubmed/?term=Plendl%20H%5BAuthor%5D&cauthor=true&cauthor_uid=25857409), [Niederacher D](https://www.ncbi.nlm.nih.gov/pubmed/?term=Niederacher%20D%5BAuthor%5D&cauthor=true&cauthor_uid=25857409), [Sutter C](https://www.ncbi.nlm.nih.gov/pubmed/?term=Sutter%20C%5BAuthor%5D&cauthor=true&cauthor_uid=25857409), [Wang-Gohrke S](https://www.ncbi.nlm.nih.gov/pubmed/?term=Wang-Gohrke%20S%5BAuthor%5D&cauthor=true&cauthor_uid=25857409), [Steinemann D](https://www.ncbi.nlm.nih.gov/pubmed/?term=Steinemann%20D%5BAuthor%5D&cauthor=true&cauthor_uid=25857409), [Preisler-Adams S](https://www.ncbi.nlm.nih.gov/pubmed/?term=Preisler-Adams%20S%5BAuthor%5D&cauthor=true&cauthor_uid=25857409), [Kast K](https://www.ncbi.nlm.nih.gov/pubmed/?term=Kast%20K%5BAuthor%5D&cauthor=true&cauthor_uid=25857409), [Varon-Mateeva R](https://www.ncbi.nlm.nih.gov/pubmed/?term=Varon-Mateeva%20R%5BAuthor%5D&cauthor=true&cauthor_uid=25857409), [Ellis S](https://www.ncbi.nlm.nih.gov/pubmed/?term=Ellis%20S%5BAuthor%5D&cauthor=true&cauthor_uid=25857409), [Frost D](https://www.ncbi.nlm.nih.gov/pubmed/?term=Frost%20D%5BAuthor%5D&cauthor=true&cauthor_uid=25857409), [Platte R](https://www.ncbi.nlm.nih.gov/pubmed/?term=Platte%20R%5BAuthor%5D&cauthor=true&cauthor_uid=25857409), [Perkins J](https://www.ncbi.nlm.nih.gov/pubmed/?term=Perkins%20J%5BAuthor%5D&cauthor=true&cauthor_uid=25857409), [Evans DG](https://www.ncbi.nlm.nih.gov/pubmed/?term=Evans%20DG%5BAuthor%5D&cauthor=true&cauthor_uid=25857409), [Izatt L](https://www.ncbi.nlm.nih.gov/pubmed/?term=Izatt%20L%5BAuthor%5D&cauthor=true&cauthor_uid=25857409), [Eeles R](https://www.ncbi.nlm.nih.gov/pubmed/?term=Eeles%20R%5BAuthor%5D&cauthor=true&cauthor_uid=25857409), [Adlard J](https://www.ncbi.nlm.nih.gov/pubmed/?term=Adlard%20J%5BAuthor%5D&cauthor=true&cauthor_uid=25857409), [Davidson R](https://www.ncbi.nlm.nih.gov/pubmed/?term=Davidson%20R%5BAuthor%5D&cauthor=true&cauthor_uid=25857409), [Cole T](https://www.ncbi.nlm.nih.gov/pubmed/?term=Cole%20T%5BAuthor%5D&cauthor=true&cauthor_uid=25857409), [Scuvera G](https://www.ncbi.nlm.nih.gov/pubmed/?term=Scuvera%20G%5BAuthor%5D&cauthor=true&cauthor_uid=25857409), [Manoukian S](https://www.ncbi.nlm.nih.gov/pubmed/?term=Manoukian%20S%5BAuthor%5D&cauthor=true&cauthor_uid=25857409), [Bonanni B](https://www.ncbi.nlm.nih.gov/pubmed/?term=Bonanni%20B%5BAuthor%5D&cauthor=true&cauthor_uid=25857409), [Mariette F](https://www.ncbi.nlm.nih.gov/pubmed/?term=Mariette%20F%5BAuthor%5D&cauthor=true&cauthor_uid=25857409), [Fortuzzi S](https://www.ncbi.nlm.nih.gov/pubmed/?term=Fortuzzi%20S%5BAuthor%5D&cauthor=true&cauthor_uid=25857409), [Viel A](https://www.ncbi.nlm.nih.gov/pubmed/?term=Viel%20A%5BAuthor%5D&cauthor=true&cauthor_uid=25857409), [Pasini B](https://www.ncbi.nlm.nih.gov/pubmed/?term=Pasini%20B%5BAuthor%5D&cauthor=true&cauthor_uid=25857409), [Papi L](https://www.ncbi.nlm.nih.gov/pubmed/?term=Papi%20L%5BAuthor%5D&cauthor=true&cauthor_uid=25857409), [Varesco L](https://www.ncbi.nlm.nih.gov/pubmed/?term=Varesco%20L%5BAuthor%5D&cauthor=true&cauthor_uid=25857409), [Balleine R](https://www.ncbi.nlm.nih.gov/pubmed/?term=Balleine%20R%5BAuthor%5D&cauthor=true&cauthor_uid=25857409), [Nathanson KL](https://www.ncbi.nlm.nih.gov/pubmed/?term=Nathanson%20KL%5BAuthor%5D&cauthor=true&cauthor_uid=25857409), [Domchek SM](https://www.ncbi.nlm.nih.gov/pubmed/?term=Domchek%20SM%5BAuthor%5D&cauthor=true&cauthor_uid=25857409), [Offitt K](https://www.ncbi.nlm.nih.gov/pubmed/?term=Offitt%20K%5BAuthor%5D&cauthor=true&cauthor_uid=25857409), [Jakubowska A](https://www.ncbi.nlm.nih.gov/pubmed/?term=Jakubowska%20A%5BAuthor%5D&cauthor=true&cauthor_uid=25857409), [Lindor N](https://www.ncbi.nlm.nih.gov/pubmed/?term=Lindor%20N%5BAuthor%5D&cauthor=true&cauthor_uid=25857409), [Thomassen M](https://www.ncbi.nlm.nih.gov/pubmed/?term=Thomassen%20M%5BAuthor%5D&cauthor=true&cauthor_uid=25857409), [Jensen UB](https://www.ncbi.nlm.nih.gov/pubmed/?term=Jensen%20UB%5BAuthor%5D&cauthor=true&cauthor_uid=25857409), [Rantala J](https://www.ncbi.nlm.nih.gov/pubmed/?term=Rantala%20J%5BAuthor%5D&cauthor=true&cauthor_uid=25857409), [Borg Å](https://www.ncbi.nlm.nih.gov/pubmed/?term=Borg%20%C3%85%5BAuthor%5D&cauthor=true&cauthor_uid=25857409), [Andrulis IL](https://www.ncbi.nlm.nih.gov/pubmed/?term=Andrulis%20IL%5BAuthor%5D&cauthor=true&cauthor_uid=25857409), [Miron A](https://www.ncbi.nlm.nih.gov/pubmed/?term=Miron%20A%5BAuthor%5D&cauthor=true&cauthor_uid=25857409), [Hansen TV](https://www.ncbi.nlm.nih.gov/pubmed/?term=Hansen%20TV%5BAuthor%5D&cauthor=true&cauthor_uid=25857409), [Caldes T](https://www.ncbi.nlm.nih.gov/pubmed/?term=Caldes%20T%5BAuthor%5D&cauthor=true&cauthor_uid=25857409), [Neuhausen SL](https://www.ncbi.nlm.nih.gov/pubmed/?term=Neuhausen%20SL%5BAuthor%5D&cauthor=true&cauthor_uid=25857409), [Toland AE](https://www.ncbi.nlm.nih.gov/pubmed/?term=Toland%20AE%5BAuthor%5D&cauthor=true&cauthor_uid=25857409), [Nevanlinna H](https://www.ncbi.nlm.nih.gov/pubmed/?term=Nevanlinna%20H%5BAuthor%5D&cauthor=true&cauthor_uid=25857409), [Montagna M](https://www.ncbi.nlm.nih.gov/pubmed/?term=Montagna%20M%5BAuthor%5D&cauthor=true&cauthor_uid=25857409), [Garber J](https://www.ncbi.nlm.nih.gov/pubmed/?term=Garber%20J%5BAuthor%5D&cauthor=true&cauthor_uid=25857409), [Godwin AK](https://www.ncbi.nlm.nih.gov/pubmed/?term=Godwin%20AK%5BAuthor%5D&cauthor=true&cauthor_uid=25857409), [Osorio A](https://www.ncbi.nlm.nih.gov/pubmed/?term=Osorio%20A%5BAuthor%5D&cauthor=true&cauthor_uid=25857409), [Factor RE](https://www.ncbi.nlm.nih.gov/pubmed/?term=Factor%20RE%5BAuthor%5D&cauthor=true&cauthor_uid=25857409), [Terry MB](https://www.ncbi.nlm.nih.gov/pubmed/?term=Terry%20MB%5BAuthor%5D&cauthor=true&cauthor_uid=25857409), [Rebbeck TR](https://www.ncbi.nlm.nih.gov/pubmed/?term=Rebbeck%20TR%5BAuthor%5D&cauthor=true&cauthor_uid=25857409), [Karlan BY](https://www.ncbi.nlm.nih.gov/pubmed/?term=Karlan%20BY%5BAuthor%5D&cauthor=true&cauthor_uid=25857409), [Southey M](https://www.ncbi.nlm.nih.gov/pubmed/?term=Southey%20M%5BAuthor%5D&cauthor=true&cauthor_uid=25857409), [Rashid MU](https://www.ncbi.nlm.nih.gov/pubmed/?term=Rashid%20MU%5BAuthor%5D&cauthor=true&cauthor_uid=25857409), [Tung N](https://www.ncbi.nlm.nih.gov/pubmed/?term=Tung%20N%5BAuthor%5D&cauthor=true&cauthor_uid=25857409), [Pharoah PD](https://www.ncbi.nlm.nih.gov/pubmed/?term=Pharoah%20PD%5BAuthor%5D&cauthor=true&cauthor_uid=25857409), [Blows FM](https://www.ncbi.nlm.nih.gov/pubmed/?term=Blows%20FM%5BAuthor%5D&cauthor=true&cauthor_uid=25857409), [Dunning AM](https://www.ncbi.nlm.nih.gov/pubmed/?term=Dunning%20AM%5BAuthor%5D&cauthor=true&cauthor_uid=25857409), [Provenzano E](https://www.ncbi.nlm.nih.gov/pubmed/?term=Provenzano%20E%5BAuthor%5D&cauthor=true&cauthor_uid=25857409), [Hall P](https://www.ncbi.nlm.nih.gov/pubmed/?term=Hall%20P%5BAuthor%5D&cauthor=true&cauthor_uid=25857409), [Czene K](https://www.ncbi.nlm.nih.gov/pubmed/?term=Czene%20K%5BAuthor%5D&cauthor=true&cauthor_uid=25857409), [Schmidt MK](https://www.ncbi.nlm.nih.gov/pubmed/?term=Schmidt%20MK%5BAuthor%5D&cauthor=true&cauthor_uid=25857409), [Broeks A](https://www.ncbi.nlm.nih.gov/pubmed/?term=Broeks%20A%5BAuthor%5D&cauthor=true&cauthor_uid=25857409), [Cornelissen S](https://www.ncbi.nlm.nih.gov/pubmed/?term=Cornelissen%20S%5BAuthor%5D&cauthor=true&cauthor_uid=25857409), [Verhoef S](https://www.ncbi.nlm.nih.gov/pubmed/?term=Verhoef%20S%5BAuthor%5D&cauthor=true&cauthor_uid=25857409), [Fasching PA](https://www.ncbi.nlm.nih.gov/pubmed/?term=Fasching%20PA%5BAuthor%5D&cauthor=true&cauthor_uid=25857409), [Beckmann MW](https://www.ncbi.nlm.nih.gov/pubmed/?term=Beckmann%20MW%5BAuthor%5D&cauthor=true&cauthor_uid=25857409), [Ekici AB](https://www.ncbi.nlm.nih.gov/pubmed/?term=Ekici%20AB%5BAuthor%5D&cauthor=true&cauthor_uid=25857409), [Slamon DJ](https://www.ncbi.nlm.nih.gov/pubmed/?term=Slamon%20DJ%5BAuthor%5D&cauthor=true&cauthor_uid=25857409), [Bojesen SE](https://www.ncbi.nlm.nih.gov/pubmed/?term=Bojesen%20SE%5BAuthor%5D&cauthor=true&cauthor_uid=25857409), [Nordestgaard BG](https://www.ncbi.nlm.nih.gov/pubmed/?term=Nordestgaard%20BG%5BAuthor%5D&cauthor=true&cauthor_uid=25857409), [Nielsen SF](https://www.ncbi.nlm.nih.gov/pubmed/?term=Nielsen%20SF%5BAuthor%5D&cauthor=true&cauthor_uid=25857409), [Flyger H](https://www.ncbi.nlm.nih.gov/pubmed/?term=Flyger%20H%5BAuthor%5D&cauthor=true&cauthor_uid=25857409), [Chang-Claude J](https://www.ncbi.nlm.nih.gov/pubmed/?term=Chang-Claude%20J%5BAuthor%5D&cauthor=true&cauthor_uid=25857409), [Flesch-Janys D](https://www.ncbi.nlm.nih.gov/pubmed/?term=Flesch-Janys%20D%5BAuthor%5D&cauthor=true&cauthor_uid=25857409), [Rudolph A](https://www.ncbi.nlm.nih.gov/pubmed/?term=Rudolph%20A%5BAuthor%5D&cauthor=true&cauthor_uid=25857409), [Seibold P](https://www.ncbi.nlm.nih.gov/pubmed/?term=Seibold%20P%5BAuthor%5D&cauthor=true&cauthor_uid=25857409), [Aittomäki K](https://www.ncbi.nlm.nih.gov/pubmed/?term=Aittom%C3%A4ki%20K%5BAuthor%5D&cauthor=true&cauthor_uid=25857409), [Muranen TA](https://www.ncbi.nlm.nih.gov/pubmed/?term=Muranen%20TA%5BAuthor%5D&cauthor=true&cauthor_uid=25857409), [Heikkilä P](https://www.ncbi.nlm.nih.gov/pubmed/?term=Heikkil%C3%A4%20P%5BAuthor%5D&cauthor=true&cauthor_uid=25857409), [Blomqvist C](https://www.ncbi.nlm.nih.gov/pubmed/?term=Blomqvist%20C%5BAuthor%5D&cauthor=true&cauthor_uid=25857409), [Figueroa J](https://www.ncbi.nlm.nih.gov/pubmed/?term=Figueroa%20J%5BAuthor%5D&cauthor=true&cauthor_uid=25857409), [Chanock SJ](https://www.ncbi.nlm.nih.gov/pubmed/?term=Chanock%20SJ%5BAuthor%5D&cauthor=true&cauthor_uid=25857409), [Brinton L](https://www.ncbi.nlm.nih.gov/pubmed/?term=Brinton%20L%5BAuthor%5D&cauthor=true&cauthor_uid=25857409), [Lissowska J](https://www.ncbi.nlm.nih.gov/pubmed/?term=Lissowska%20J%5BAuthor%5D&cauthor=true&cauthor_uid=25857409), [Olson JE](https://www.ncbi.nlm.nih.gov/pubmed/?term=Olson%20JE%5BAuthor%5D&cauthor=true&cauthor_uid=25857409), [Pankratz VS](https://www.ncbi.nlm.nih.gov/pubmed/?term=Pankratz%20VS%5BAuthor%5D&cauthor=true&cauthor_uid=25857409), [John EM](https://www.ncbi.nlm.nih.gov/pubmed/?term=John%20EM%5BAuthor%5D&cauthor=true&cauthor_uid=25857409), [Whittemore AS](https://www.ncbi.nlm.nih.gov/pubmed/?term=Whittemore%20AS%5BAuthor%5D&cauthor=true&cauthor_uid=25857409), [West DW](https://www.ncbi.nlm.nih.gov/pubmed/?term=West%20DW%5BAuthor%5D&cauthor=true&cauthor_uid=25857409), [Hamann U](https://www.ncbi.nlm.nih.gov/pubmed/?term=Hamann%20U%5BAuthor%5D&cauthor=true&cauthor_uid=25857409), [Torres D](https://www.ncbi.nlm.nih.gov/pubmed/?term=Torres%20D%5BAuthor%5D&cauthor=true&cauthor_uid=25857409), [Ulmer HU](https://www.ncbi.nlm.nih.gov/pubmed/?term=Ulmer%20HU%5BAuthor%5D&cauthor=true&cauthor_uid=25857409), [Rüdiger T](https://www.ncbi.nlm.nih.gov/pubmed/?term=R%C3%BCdiger%20T%5BAuthor%5D&cauthor=true&cauthor_uid=25857409), [Devilee P](https://www.ncbi.nlm.nih.gov/pubmed/?term=Devilee%20P%5BAuthor%5D&cauthor=true&cauthor_uid=25857409), [Tollenaar RA](https://www.ncbi.nlm.nih.gov/pubmed/?term=Tollenaar%20RA%5BAuthor%5D&cauthor=true&cauthor_uid=25857409), [Seynaeve C](https://www.ncbi.nlm.nih.gov/pubmed/?term=Seynaeve%20C%5BAuthor%5D&cauthor=true&cauthor_uid=25857409), [Van Asperen CJ](https://www.ncbi.nlm.nih.gov/pubmed/?term=Van%20Asperen%20CJ%5BAuthor%5D&cauthor=true&cauthor_uid=25857409), [Eccles DM](https://www.ncbi.nlm.nih.gov/pubmed/?term=Eccles%20DM%5BAuthor%5D&cauthor=true&cauthor_uid=25857409), [Tapper WJ](https://www.ncbi.nlm.nih.gov/pubmed/?term=Tapper%20WJ%5BAuthor%5D&cauthor=true&cauthor_uid=25857409), [Durcan L](https://www.ncbi.nlm.nih.gov/pubmed/?term=Durcan%20L%5BAuthor%5D&cauthor=true&cauthor_uid=25857409), [Jones L](https://www.ncbi.nlm.nih.gov/pubmed/?term=Jones%20L%5BAuthor%5D&cauthor=true&cauthor_uid=25857409), [Peto J](https://www.ncbi.nlm.nih.gov/pubmed/?term=Peto%20J%5BAuthor%5D&cauthor=true&cauthor_uid=25857409), [dos-Santos-Silva I](https://www.ncbi.nlm.nih.gov/pubmed/?term=dos-Santos-Silva%20I%5BAuthor%5D&cauthor=true&cauthor_uid=25857409), [Fletcher O](https://www.ncbi.nlm.nih.gov/pubmed/?term=Fletcher%20O%5BAuthor%5D&cauthor=true&cauthor_uid=25857409), [Johnson N](https://www.ncbi.nlm.nih.gov/pubmed/?term=Johnson%20N%5BAuthor%5D&cauthor=true&cauthor_uid=25857409), [Dwek M](https://www.ncbi.nlm.nih.gov/pubmed/?term=Dwek%20M%5BAuthor%5D&cauthor=true&cauthor_uid=25857409), [Swann R](https://www.ncbi.nlm.nih.gov/pubmed/?term=Swann%20R%5BAuthor%5D&cauthor=true&cauthor_uid=25857409), [Bane AL](https://www.ncbi.nlm.nih.gov/pubmed/?term=Bane%20AL%5BAuthor%5D&cauthor=true&cauthor_uid=25857409), [Glendon G](https://www.ncbi.nlm.nih.gov/pubmed/?term=Glendon%20G%5BAuthor%5D&cauthor=true&cauthor_uid=25857409), [Mulligan AM](https://www.ncbi.nlm.nih.gov/pubmed/?term=Mulligan%20AM%5BAuthor%5D&cauthor=true&cauthor_uid=25857409), [Giles GG](https://www.ncbi.nlm.nih.gov/pubmed/?term=Giles%20GG%5BAuthor%5D&cauthor=true&cauthor_uid=25857409), [Milne RL](https://www.ncbi.nlm.nih.gov/pubmed/?term=Milne%20RL%5BAuthor%5D&cauthor=true&cauthor_uid=25857409), [Baglietto L](https://www.ncbi.nlm.nih.gov/pubmed/?term=Baglietto%20L%5BAuthor%5D&cauthor=true&cauthor_uid=25857409), [McLean C](https://www.ncbi.nlm.nih.gov/pubmed/?term=McLean%20C%5BAuthor%5D&cauthor=true&cauthor_uid=25857409), [Carpenter J](https://www.ncbi.nlm.nih.gov/pubmed/?term=Carpenter%20J%5BAuthor%5D&cauthor=true&cauthor_uid=25857409), [Clarke C](https://www.ncbi.nlm.nih.gov/pubmed/?term=Clarke%20C%5BAuthor%5D&cauthor=true&cauthor_uid=25857409), [Scott R](https://www.ncbi.nlm.nih.gov/pubmed/?term=Scott%20R%5BAuthor%5D&cauthor=true&cauthor_uid=25857409), [Brauch H](https://www.ncbi.nlm.nih.gov/pubmed/?term=Brauch%20H%5BAuthor%5D&cauthor=true&cauthor_uid=25857409), [Brüning T](https://www.ncbi.nlm.nih.gov/pubmed/?term=Br%C3%BCning%20T%5BAuthor%5D&cauthor=true&cauthor_uid=25857409), [Ko YD](https://www.ncbi.nlm.nih.gov/pubmed/?term=Ko%20YD%5BAuthor%5D&cauthor=true&cauthor_uid=25857409), [Cox A](https://www.ncbi.nlm.nih.gov/pubmed/?term=Cox%20A%5BAuthor%5D&cauthor=true&cauthor_uid=25857409), [Cross SS](https://www.ncbi.nlm.nih.gov/pubmed/?term=Cross%20SS%5BAuthor%5D&cauthor=true&cauthor_uid=25857409), [Reed MW](https://www.ncbi.nlm.nih.gov/pubmed/?term=Reed%20MW%5BAuthor%5D&cauthor=true&cauthor_uid=25857409), [Lubinski J](https://www.ncbi.nlm.nih.gov/pubmed/?term=Lubinski%20J%5BAuthor%5D&cauthor=true&cauthor_uid=25857409), [Jaworska-Bieniek K](https://www.ncbi.nlm.nih.gov/pubmed/?term=Jaworska-Bieniek%20K%5BAuthor%5D&cauthor=true&cauthor_uid=25857409), [Durda K](https://www.ncbi.nlm.nih.gov/pubmed/?term=Durda%20K%5BAuthor%5D&cauthor=true&cauthor_uid=25857409), [Gronwald J](https://www.ncbi.nlm.nih.gov/pubmed/?term=Gronwald%20J%5BAuthor%5D&cauthor=true&cauthor_uid=25857409), [Dörk T](https://www.ncbi.nlm.nih.gov/pubmed/?term=D%C3%B6rk%20T%5BAuthor%5D&cauthor=true&cauthor_uid=25857409), [Bogdanova N](https://www.ncbi.nlm.nih.gov/pubmed/?term=Bogdanova%20N%5BAuthor%5D&cauthor=true&cauthor_uid=25857409), [Park-Simon TW](https://www.ncbi.nlm.nih.gov/pubmed/?term=Park-Simon%20TW%5BAuthor%5D&cauthor=true&cauthor_uid=25857409), [Hillemanns P](https://www.ncbi.nlm.nih.gov/pubmed/?term=Hillemanns%20P%5BAuthor%5D&cauthor=true&cauthor_uid=25857409), [Haiman CA](https://www.ncbi.nlm.nih.gov/pubmed/?term=Haiman%20CA%5BAuthor%5D&cauthor=true&cauthor_uid=25857409), [Henderson BE](https://www.ncbi.nlm.nih.gov/pubmed/?term=Henderson%20BE%5BAuthor%5D&cauthor=true&cauthor_uid=25857409), [Schumacher F](https://www.ncbi.nlm.nih.gov/pubmed/?term=Schumacher%20F%5BAuthor%5D&cauthor=true&cauthor_uid=25857409), [Le Marchand L](https://www.ncbi.nlm.nih.gov/pubmed/?term=Le%20Marchand%20L%5BAuthor%5D&cauthor=true&cauthor_uid=25857409), [Burwinkel B](https://www.ncbi.nlm.nih.gov/pubmed/?term=Burwinkel%20B%5BAuthor%5D&cauthor=true&cauthor_uid=25857409), [Marme F](https://www.ncbi.nlm.nih.gov/pubmed/?term=Marme%20F%5BAuthor%5D&cauthor=true&cauthor_uid=25857409), [Surovy H](https://www.ncbi.nlm.nih.gov/pubmed/?term=Surovy%20H%5BAuthor%5D&cauthor=true&cauthor_uid=25857409), [Yang R](https://www.ncbi.nlm.nih.gov/pubmed/?term=Yang%20R%5BAuthor%5D&cauthor=true&cauthor_uid=25857409), [Anton-Culver H](https://www.ncbi.nlm.nih.gov/pubmed/?term=Anton-Culver%20H%5BAuthor%5D&cauthor=true&cauthor_uid=25857409), [Ziogas A](https://www.ncbi.nlm.nih.gov/pubmed/?term=Ziogas%20A%5BAuthor%5D&cauthor=true&cauthor_uid=25857409), [Hooning MJ](https://www.ncbi.nlm.nih.gov/pubmed/?term=Hooning%20MJ%5BAuthor%5D&cauthor=true&cauthor_uid=25857409), [Collée JM](https://www.ncbi.nlm.nih.gov/pubmed/?term=Coll%C3%A9e%20JM%5BAuthor%5D&cauthor=true&cauthor_uid=25857409), [Martens JW](https://www.ncbi.nlm.nih.gov/pubmed/?term=Martens%20JW%5BAuthor%5D&cauthor=true&cauthor_uid=25857409), [Tilanus-Linthorst MM](https://www.ncbi.nlm.nih.gov/pubmed/?term=Tilanus-Linthorst%20MM%5BAuthor%5D&cauthor=true&cauthor_uid=25857409), [Brenner H](https://www.ncbi.nlm.nih.gov/pubmed/?term=Brenner%20H%5BAuthor%5D&cauthor=true&cauthor_uid=25857409), [Dieffenbach AK](https://www.ncbi.nlm.nih.gov/pubmed/?term=Dieffenbach%20AK%5BAuthor%5D&cauthor=true&cauthor_uid=25857409), [Arndt V](https://www.ncbi.nlm.nih.gov/pubmed/?term=Arndt%20V%5BAuthor%5D&cauthor=true&cauthor_uid=25857409), [Stegmaier C](https://www.ncbi.nlm.nih.gov/pubmed/?term=Stegmaier%20C%5BAuthor%5D&cauthor=true&cauthor_uid=25857409), [Winqvist R](https://www.ncbi.nlm.nih.gov/pubmed/?term=Winqvist%20R%5BAuthor%5D&cauthor=true&cauthor_uid=25857409), [Pylkäs K](https://www.ncbi.nlm.nih.gov/pubmed/?term=Pylk%C3%A4s%20K%5BAuthor%5D&cauthor=true&cauthor_uid=25857409), [Jukkola-Vuorinen A](https://www.ncbi.nlm.nih.gov/pubmed/?term=Jukkola-Vuorinen%20A%5BAuthor%5D&cauthor=true&cauthor_uid=25857409), [Grip M](https://www.ncbi.nlm.nih.gov/pubmed/?term=Grip%20M%5BAuthor%5D&cauthor=true&cauthor_uid=25857409), [Lindblom A](https://www.ncbi.nlm.nih.gov/pubmed/?term=Lindblom%20A%5BAuthor%5D&cauthor=true&cauthor_uid=25857409), [Margolin S](https://www.ncbi.nlm.nih.gov/pubmed/?term=Margolin%20S%5BAuthor%5D&cauthor=true&cauthor_uid=25857409), [Joseph V](https://www.ncbi.nlm.nih.gov/pubmed/?term=Joseph%20V%5BAuthor%5D&cauthor=true&cauthor_uid=25857409), [Robson M](https://www.ncbi.nlm.nih.gov/pubmed/?term=Robson%20M%5BAuthor%5D&cauthor=true&cauthor_uid=25857409), [Rau-Murthy R](https://www.ncbi.nlm.nih.gov/pubmed/?term=Rau-Murthy%20R%5BAuthor%5D&cauthor=true&cauthor_uid=25857409), [González-Neira A](https://www.ncbi.nlm.nih.gov/pubmed/?term=Gonz%C3%A1lez-Neira%20A%5BAuthor%5D&cauthor=true&cauthor_uid=25857409), [Arias JI](https://www.ncbi.nlm.nih.gov/pubmed/?term=Arias%20JI%5BAuthor%5D&cauthor=true&cauthor_uid=25857409), [Zamora P](https://www.ncbi.nlm.nih.gov/pubmed/?term=Zamora%20P%5BAuthor%5D&cauthor=true&cauthor_uid=25857409), [Benítez J](https://www.ncbi.nlm.nih.gov/pubmed/?term=Ben%C3%ADtez%20J%5BAuthor%5D&cauthor=true&cauthor_uid=25857409), [Mannermaa A](https://www.ncbi.nlm.nih.gov/pubmed/?term=Mannermaa%20A%5BAuthor%5D&cauthor=true&cauthor_uid=25857409), [Kataja V](https://www.ncbi.nlm.nih.gov/pubmed/?term=Kataja%20V%5BAuthor%5D&cauthor=true&cauthor_uid=25857409), [Kosma VM](https://www.ncbi.nlm.nih.gov/pubmed/?term=Kosma%20VM%5BAuthor%5D&cauthor=true&cauthor_uid=25857409), [Hartikainen JM](https://www.ncbi.nlm.nih.gov/pubmed/?term=Hartikainen%20JM%5BAuthor%5D&cauthor=true&cauthor_uid=25857409), [Peterlongo P](https://www.ncbi.nlm.nih.gov/pubmed/?term=Peterlongo%20P%5BAuthor%5D&cauthor=true&cauthor_uid=25857409), [Zaffaroni D](https://www.ncbi.nlm.nih.gov/pubmed/?term=Zaffaroni%20D%5BAuthor%5D&cauthor=true&cauthor_uid=25857409), [Barile M](https://www.ncbi.nlm.nih.gov/pubmed/?term=Barile%20M%5BAuthor%5D&cauthor=true&cauthor_uid=25857409), [Capra F](https://www.ncbi.nlm.nih.gov/pubmed/?term=Capra%20F%5BAuthor%5D&cauthor=true&cauthor_uid=25857409), [Radice P](https://www.ncbi.nlm.nih.gov/pubmed/?term=Radice%20P%5BAuthor%5D&cauthor=true&cauthor_uid=25857409), [Teo SH](https://www.ncbi.nlm.nih.gov/pubmed/?term=Teo%20SH%5BAuthor%5D&cauthor=true&cauthor_uid=25857409), [Easton DF](https://www.ncbi.nlm.nih.gov/pubmed/?term=Easton%20DF%5BAuthor%5D&cauthor=true&cauthor_uid=25857409), [Antoniou AC](https://www.ncbi.nlm.nih.gov/pubmed/?term=Antoniou%20AC%5BAuthor%5D&cauthor=true&cauthor_uid=25857409), [Chenevix-Trench G](https://www.ncbi.nlm.nih.gov/pubmed/?term=Chenevix-Trench%20G%5BAuthor%5D&cauthor=true&cauthor_uid=25857409), [Goldgar DE](https://www.ncbi.nlm.nih.gov/pubmed/?term=Goldgar%20DE%5BAuthor%5D&cauthor=true&cauthor_uid=25857409); [ABCTB Investigators](https://www.ncbi.nlm.nih.gov/pubmed/?term=ABCTB%20Investigators%5BCorporate%20Author%5D); [EMBRACE Group](https://www.ncbi.nlm.nih.gov/pubmed/?term=EMBRACE%20Group%5BCorporate%20Author%5D); [GENICA Network](https://www.ncbi.nlm.nih.gov/pubmed/?term=GENICA%20Network%5BCorporate%20Author%5D); [HEBON Group](https://www.ncbi.nlm.nih.gov/pubmed/?term=HEBON%20Group%5BCorporate%20Author%5D); [kConFab Investigators](https://www.ncbi.nlm.nih.gov/pubmed/?term=kConFab%20Investigators%5BCorporate%20Author%5D). 2014. Refined histopathological predictors of BRCA1 and BRCA2 mutation status: a large-scale analysis of breast cancer characteristics from the BCAC, CIMBA, and ENIGMA consortia. Breast Cancer Res 16(6):3419.

Thompson D, Easton DF, Goldgar DE. 2003. A full-likelihood method for the evaluation of causality of sequence variants from family data. Am J Hum Genet 73:652-655.

Vallee MP, Di Sera TL, Nix DA, Paquette AM, Parsons MT, Bell R, Hoffman A, Hogervorst FB, Goldgar DE, Spurdle AB, Tavtigian SV. 2016. Adding In Silico Assessment of Potential Splice Aberration to the Integrated Evaluation of BRCA Gene Unclassified Variants. Hum Mutat 37(7):627-639.

**Supplementary Figure S1. *BRCA2* exon 3 exclusion and inclusion rates in six c.68-7T>A carriers and 12 normal controls. The bars represent qPCR and dPCR measures of exclusion rate (see Materials and Methods) and inclusion rate (100-exclusion rate).**

**Supplementary Figure S2. Growth inhibition assay of *BRCA2* deficient fibroblasts (EUFA423, CAPAN1) and LCLs exposed to Mitomycin C (MMC).** Data are the average of three independent experiments. Error bars represent the standard error of the mean. Statistically significant differences (p<0.01) in the level of viability between MMC-treated and untreated cells are indicated by asterisks (mut, *BRCA2* c.5722_5723delCT; VUS, *BRCA2* c.68-7 T>A).

**Supplementary Figure S3. Inter-individual variability of the total amount of *BRCA2* transcripts in carriers of the c.68-7T>A variant and normal controls. The bars represent the total amount of *BRCA2* transcripts in each sample obtained by summing up the amount of ▼3 and Δ3 transcripts, and setting as 1 the average total expression observed in our cohort. The inter-individual variability ranges from 0.43 to 1.50, with many control samples clustering above the average.**

**Supplementary Table S1.** List of primers for each assays

| **Assay** | **Forward Primer** | |  | **Reverse Primer** | | **Amplicon Size** |
| --- | --- | --- | --- | --- | --- | --- |
|  | **Location** | **Sequence** |  | **Location** | **Sequence** |  |
| Capillary Electrophoresis − *β2M* cDNA | exon 1 | 5′-ATATAAGTGGAGGCGTCG-3′ |  | exon 1-2 junction | 5′-(GTTTCTT)AGTCACATGGTTCACACG-3′ | 377bp |
| Capillary Electrophoresis − *BRCA2* cDNA | 5’-UTR | 5′-GAGGGGACAGATTTGTGACC-3′ |  | exon 4 | 5′-GTCTACTATTGGGAACATTCC-3′ | 454^#^/205^§^bp |
| Allelic Specific Expression −▼3 transcript | 5’-UTR | 5′-gccgggagaagcgtgagggg-3′ |  | exon 3 | 5′-ggagttgaagccagctgat-3′ | 361bp |
| Allelic Specific Expression − ∆3 transcript | 5’-UTR | 5′-gccgggagaagcgtgagggg-3′ |  | exon 2-4 junction | 5′-ATTGGGAACATTCCTTCCTG-3′ | 212bp |
| qPCR − *GUSB* | exon 2 | 5′-AGAGTGGTGCTGAGGATTGG-3′ |  | exon 3 | 5′-CCCTCATGCTCTAGCGTGTC-3′ | 80bp |
| qPCR − Δ3 transcript | exon 2-4 junction | 5′-GCTGCAACAAAGCAGGAA-3′ |  | exon 4 | 5′-AAGAATTTAGAAGTGGACAGG-3′ | 111bp |
| qPCR −▼3 transcript | exon 2 | 5′-gagaggccaacattttttg-3′ |  | exon 3 | 5′-ggagcttctgaagaaagttc-3′ | 104bp |

The forward primers used in Capillary Electrophoresis were labeled at the 5′-end with Hexachloro-6-carboxy-fluorescein (HEX) for *β2M* cDNA assay and with 6-carboxy-fluorescein (6-FAM) for *BRCA2* cDNA assay. The reverse primer used to amplify the *β2M* cDNA fragment for Capillary Electrophoresis analysis contains a sequence of 7 bases in order to overcome the problems associated with nontemplated nucleotide addition.  ^#^size of the amplicon corresponding to the isoforms retaining the exon 3; ^§^size of the amplicon corresponding to the isoforms missing the exon 3.

**Supplementary Table S2.** *BRCA2* c.68-7T>A positive individuals with *in trans* pathogenic variants

| **Source** | **Study** | **Family ID** | **Case ID** | ***BRCA2* Mutation**  **(HGVS-nomenclature)** |
| --- | --- | --- | --- | --- |
|  |  |  |  |  |
| CIMBA | BCFR- Ontario | F1 | 165704 | c.1813delA |
| CIMBA | BMBSA | F2 | 193070  (SA) | c.7934delG |
| CIMBA | HEBON Center 2 | F3 | 165296 | c.9672dupA |
| CIMBA | EMBRACE Center 5 | F4 | 178543 | c.8633-?_9256+?del |
| CIMBA | EMBRACE Center 7 | F5 | 178473 | c.8904delC |
| CIMBA | EMBRACE Center 7 | F5 | 178474 | c.8904delC |
| CIMBA | EMBRACE Center 20 | F6 | 178993 | c.5946delT |
| CIMBA | KconFab | F7 | 163662 | c.6275_6276delTT |
| CIMBA | KconFab | F8 | 163645 | c.-227-?_67+?del |
| CIMBA | KconFab | F8 | 163657 | c.-227-?_67+?del |
| CIMBA | MSKCC | F9 | 167160 | c.5946delT |
| CIMBA | MSKCC | F10 | 165570 | c.5946delT |
| CIMBA | OCGN | F11 | 165787 | c.5946delT |
| ENIGMA | IOV | F12 | 304.1.21 | c.6447_6448dupTA |
| INT | INT | F13 | M28260001 | c.5722_5723delCT |
